# Direct Observation of Ammonia Storage in UiO-66 Incorporating Cu(II) Binding Sites

**DOI:** 10.1021/jacs.2c00952

**Published:** 2022-05-09

**Authors:** Yujie Ma, Wanpeng Lu, Xue Han, Yinlin Chen, Ivan da Silva, Daniel Lee, Alena M. Sheveleva, Zi Wang, Jiangnan Li, Weiyao Li, Mengtian Fan, Shaojun Xu, Floriana Tuna, Eric J. L. McInnes, Yongqiang Cheng, Svemir Rudić, Pascal Manuel, Mark D. Frogley, Anibal J. Ramirez-Cuesta, Martin Schröder, Sihai Yang

**Affiliations:** † Department of Chemistry, University of Manchester, Manchester M13 9PL, U.K.; ‡ ISIS Facility, Science and Technology Facilities Council, Rutherford Appleton Laboratory, Chilton OX11 0QX, U.K.; § Department of Chemical Engineering and Analytical Science, University of Manchester, Manchester M13 9PL, U.K.; ∥ Photon Science Institute, University of Manchester, Manchester M13 9PL, U.K.; ⊥ Neutron Scattering Division, Neutron Sciences Directorate, Oak Ridge National Laboratory, Oak Ridge, Tennessee 37831, United States; # Diamond Light Source, Harwell Science Campus, Oxfordshire OX11 0DE, U.K.; ∇ UK Catalysis Hub, Research Complex at Harwell, Rutherford Appleton Laboratory, Harwell OX11 0FA, U.K.; ○ School of Chemistry, Cardiff University, Cardiff CF10 3AT, U.K.

## Abstract

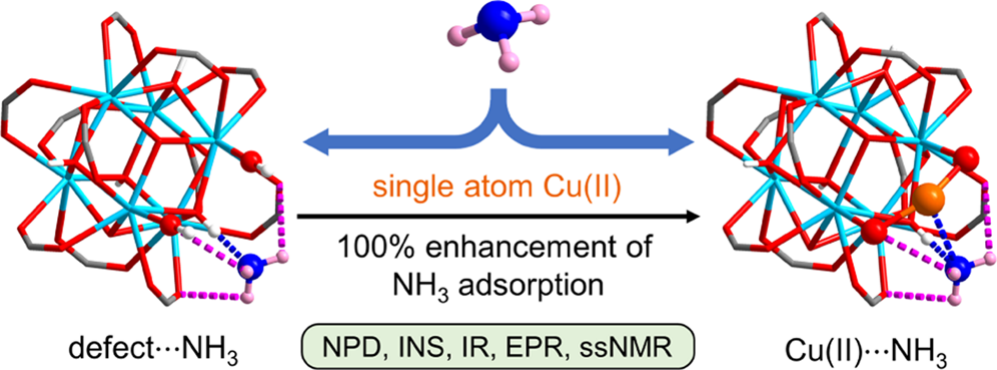

The presence of active sites in metal–organic framework (MOF) materials can control and affect their performance significantly in adsorption and catalysis. However, revealing the interactions between the substrate and active sites in MOFs at atomic precision remains a challenging task. Here, we report the direct observation of binding of NH_3_ in a series of UiO-66 materials containing atomically dispersed defects and open Cu(I) and Cu(II) sites. While all MOFs in this series exhibit similar surface areas (1111–1135 m^2^ g^–1^), decoration of the −OH site in UiO-66-defect with Cu(II) results in a 43% enhancement of the isothermal uptake of NH_3_ at 273 K and 1.0 bar from 11.8 in UiO-66-defect to 16.9 mmol g^–1^ in UiO-66-Cu^II^. A 100% enhancement of dynamic adsorption of NH_3_ at a concentration level of 630 ppm from 2.07 mmol g^–1^ in UiO-66-defect to 4.15 mmol g^–1^ in UiO-66-Cu^II^ at 298 K is observed. *In situ* neutron powder diffraction, inelastic neutron scattering, and electron paramagnetic resonance, solid-state nuclear magnetic resonance, and infrared spectroscopies, coupled with modeling reveal that the enhanced NH_3_ uptake in UiO-66-Cu^II^ originates from a {Cu(II)···NH_3_} interaction, with a reversible change in geometry at Cu(II) from near-linear to trigonal coordination. This work represents the first example of structural elucidation of NH_3_ binding in MOFs containing open metal sites and will inform the design of new efficient MOF sorbents by targeted control of active sites for NH_3_ capture and storage.

## Introduction

Ammonia (NH_3_) is a major feedstock in the agricultural and chemical industries,^1^ but due to its toxic and corrosive nature, storage and transport of NH_3_ in large quantities is challenging.^2^ It is therefore of great interest to develop efficient sorbent materials that show significant chemical and physical stability and high adsorption capacity for NH_3_. Conventional sorbents, including zeolites,^3^ activated carbons,^4^ mesoporous silica,^5^ and resins,^6^ have been studied for NH_3_ adsorption, but they show limited capacities and often undergo irreversible structural degradation upon desorption. In addition, fine-tuning and directed chemical manipulation of active sites in these materials at the atomic level can be problematic due to the lack of direct structural insights and limited structural diversity.

Porous metal–organic framework (MOF) materials adopt well-defined structures, are designable, and can show exceptional structural diversity, enabling the control of active sites at atomic precision.^7−10^ Structural defects and open metal sites in MOFs are widely considered and used as active sites for binding of guest molecules.^11−18^ A variety of MOFs incorporating open metal sites or defects, such as [M_2_(dobpdc)] (dobpdc^4**–**^ = 4,4-dioxidobi-phenyl-3,3-dicarboxylate),^2^ Cu_2_Cl_2_(BBTA) [BBTA = 1*H*,5*H*-benzo(1,2-*d*),(4,5-*d′*)bistriazole],^19^ HKUST-1,^20^ and UiO-67,^21^ have been reported for NH_3_ adsorption. However, it remains highly challenging to identify the precise role of these active sites in binding NH_3_ molecules, not least because of the relative invisibility of protons in NH_3_ by X-ray diffraction and the complex and rapid host–guest dynamics involved in NH_3_ binding. Revealing such insights will enable targeted control of active sites and thus deliver efficient NH_3_ stores by design. This will further inform the development of next-generation catalysts for the cracking of NH_3_ for portable applications relating to the hydrogen economy.

Here, we report the study of binding domains and dynamics of NH_3_ within UiO-66-defect (UiO-66 with a missing carboxylate ligand), UiO-66-Cu^I^, and UiO-66-Cu^II^ based upon the direct observation of the location of atomically dispersed active sites and their interactions with NH_3_ molecules. The robustness of the framework in UiO-66 makes it an ideal platform for the study of NH_3_ adsorption, and the incorporation of open Cu(II) sites can provide further strong binding and activation sites. Compared with UiO-66-defect, UiO-66-Cu^II^ shows significant enhancement of static (11.8 and 16.9 mmol g^–1^, respectively, at 273 K and 1.0 bar) and dynamic (2.07 and 4.15 mmol g^–1^, respectively, at 298 K and at 630 ppm concentrations) adsorption of NH_3_, thus serving as a top-performing NH_3_ sorbent. *In situ* neutron powder diffraction (NPD), inelastic neutron scattering (INS), coupled with density functional theory (DFT) modeling, electron paramagnetic resonance (EPR), solid-state nuclear magnetic resonance (ssNMR), infrared (IR), and ultraviolet–visible (UV–vis) absorption spectroscopies reveal the presence of reversible {Cu(II)···NH_3_} interactions that underpin the observed high and reversible NH_3_ uptake.

## Experimental Section

### NH_3_ Adsorption Isotherms and Cycling Experiment

The synthesis and activation of MOF materials have been reported in our previous study^22^ and are described in detail in the Supporting Information. Static adsorption isotherms (0–1.0 bar) for NH_3_ were measured on IGA (intelligent gravimetric analyzer, Hiden Isochema, Warrington, U.K.). Desolvated samples of UiO-66-defect, UiO-66-Cu^I^, and UiO-66-Cu^II^ were generated *in situ* under dynamic vacuum (1 × 10^–8^ mbar) at 393 K for 24 h. NH_3_ (research-grade) was purchased from BOC and used as received. For cycling experiments, the pressure of NH_3_ was increased from vacuum (1 × 10^–8^ mbar) to 150 mbar and the uptake was recorded. The pressure was then reduced to regenerate the sample with no assisted heating. This cycling process was repeated for 15 cycles.

### Neutron Powder Diffraction (NPD)

The binding positions of ND_3_ within UiO-66-defect and UiO-66-Cu^II^ were determined by NPD experiments at WISH, a long-wavelength powder and single-crystal neutron diffractometer at the ISIS Facility at the Rutherford Appleton Laboratory (U.K.).^23^ Prior to NPD measurements, the sample was activated by heating at 393 K under dynamic vacuum for 16 h, and the desolvated samples were then transferred into cylindrical vanadium sample cells with an indium seal. The samples were further degassed at 373 K under dynamic vacuum to remove the remaining trace guest water molecules. Dosing of ND_3_ was carried out volumetrically at room temperature to ensure that ND_3_ was present in the gas phase when not adsorbed and to ensure sufficient mobility of ND_3_ inside the MOF framework. The temperature during data collection was controlled using a helium (He) cryostat (7 ± 0.2 K). The quality of the Rietveld refinements has been assured with low goodness-of-fit (Gof) factors, low weighted profile factors (*R*_wp_), and well-fitted patterns with reasonable isotropic displacement factors.

### Inelastic Neutron Scattering (INS)

INS experiments were performed at TOSCA neutron spectrometer at the ISIS Facility at the Rutherford Appleton Laboratory (U.K.).^24^ Desolvated UiO-66-defect and UiO-66-Cu^II^ materials were loaded into cylindrical vanadium sample cells with an indium seal and degassed at 373 K under dynamic vacuum to remove the remaining trace guest water molecules. The temperature during data collection was controlled using a He cryostat (7 ± 0.2 K). The loading of NH_3_ was performed volumetrically at room temperature, and background spectra of bare MOF samples were subtracted to obtain the difference spectra.

### Solid-State Nuclear Magnetic Resonance (ssNMR) Spectroscopy

Magic angle spinning (MAS) NMR spectra were recorded using a Bruker 9.4 T (400 MHz ^1^H Larmor frequency) AVANCE III spectrometer equipped with a 4 mm HFX MAS probe. Samples were desolvated and packed into 4 mm o.d. zirconia rotors under inert conditions and sealed with a Kel-F rotor cap. Experiments were carried out at ambient temperature using a MAS frequency of 12 kHz. ^1^H-pulses of 100 kHz and ^13^C-pulses of 50 kHz were used, and ^13^C spin-locking at ∼50 kHz was applied for 2 ms, with corresponding ramped (70–100%) ^1^H spin-locking at ∼73 kHz for CP experiments and with 100 kHz of SPINAL-64^25^ heteronuclear ^1^H decoupling throughout signal acquisition. Then, 640–8192 transients were co-added for the CPMAS NMR spectra, depending on the sample. ^1^H Hahn echo spectra used an inter-pulse delay of one rotor period, giving a total echo time of 0.167 ms. For the two-dimensional (2D) ^1^H–^13^C FSLG-HETCOR^26^ dipolar correlation experiment, 2304 transients were acquired for each of 32 complex *t*_1_ increments and a CP contact time of 0.5 ms was employed.

### Electron Paramagnetic Resonance (EPR) Spectroscopy

CW EPR spectra were measured with a Bruker EMX 300 EPR spectrometer equipped with a high sensitivity X-band (*ca.* 9.4 GHz) resonator and a liquid He cryostat. The spectra were recorded at a microwave power of 0.0022–2.2 mW, modulation frequency 100 kHz, and modulation amplitude 5 G. Field corrections were applied by measuring relevant EPR standards (Bruker Strong Pitch and DPPH). Pulsed EPR measurements were performed at X-band (*ca.* 9.7 GHz) on a Bruker ElexSys E580 spectrometer. The microwave frequency was measured with a built-in digital counter, and the magnetic field was calibrated using a Bruker strong pitch reference sample.

## Results and Discussion

### NH_3_ Adsorption Analysis

**Figure 1:**
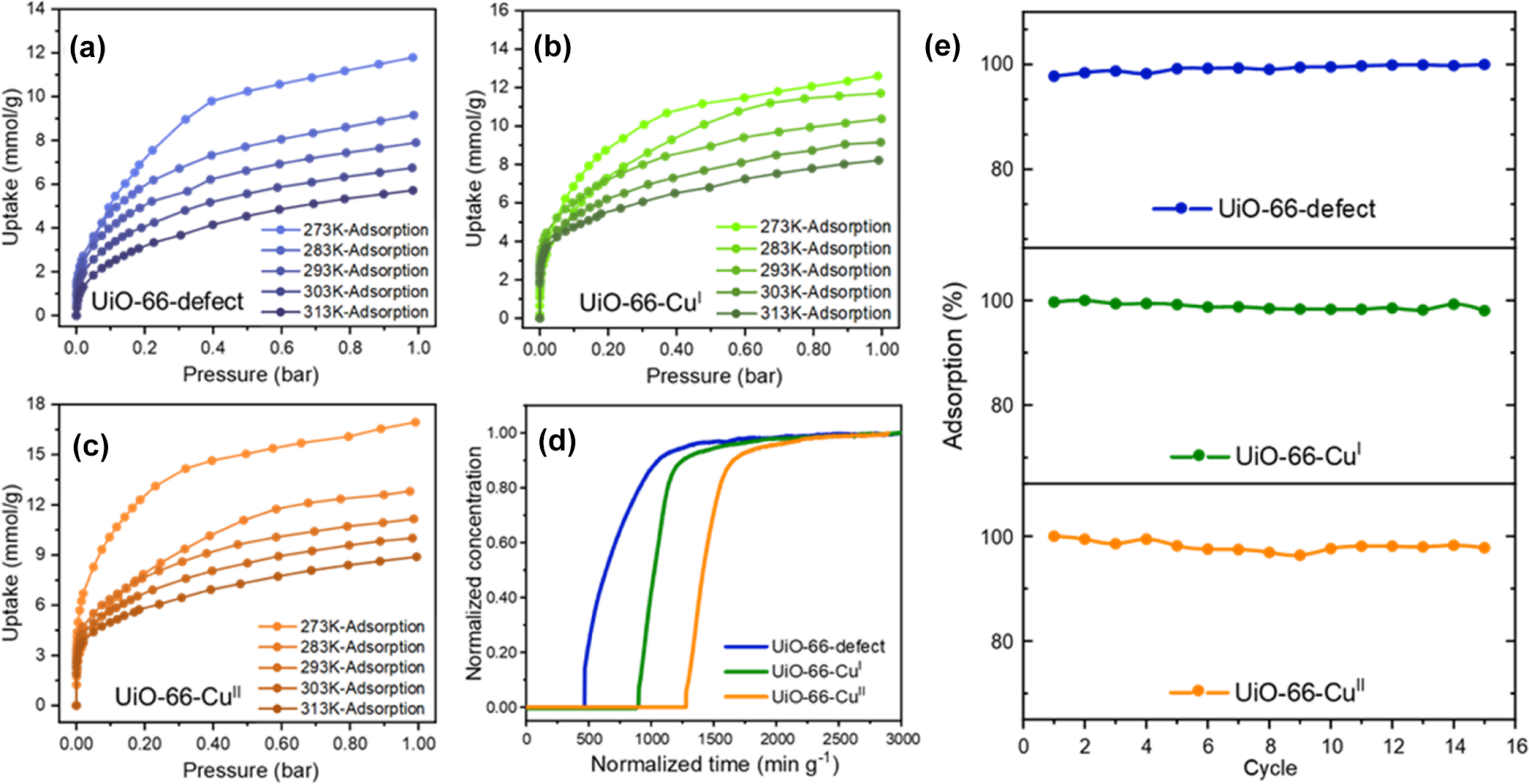
Adsorption isotherms for NH_3_ in (a) UiO-66-defect, (b) UiO-66-Cu^I^, and (c) UiO-66-Cu^II^ from 273 to 313 K. (d) Breakthrough curves at 298 K of NH_3_ (630 ppm of NH_3_ diluted in He) through a fixed-bed packed with UiO-66-defect, UiO-66-Cu^I^, and UiO-66-Cu^II^. (e) Cycles of pressure-swing sorption of NH_3_ at 298 K between 0 and 0.15 bar in UiO-66-defect, UiO-66-Cu^I^, and UiO-66-Cu^II^.

Desolvated UiO-66-defect, UiO-66-Cu^I^, and UiO-66-Cu^II^ display BET surface areas of 1135, 1111, and 1124 m^2^ g^–1^, respectively (Table S1). Thus, the variation of active sites in the pore interior has little impact on the porosity of resultant UiO-66 materials. X-ray absorption fine structure (XAFS) spectroscopy of UiO-66-Cu^II^ (Figure S2) shows a lower intensity for the features at a long distance (∼2.5 Å) in the Fourier transform of the *k*^2^-weighted data compared with that for CuO as a reference material. This strongly suggests that the Cu(II) sites in UiO-66-Cu^II^ are atomically dispersed,^22,27^ in full agreement with the EPR spectroscopic results, which confirmed the absence of aggregated (long-range magnetically coupled) or binuclear (*S* = 1) Cu(II) species in UiO-66-Cu^II^.^22^ At 273 K and 1.0 bar, UiO-66-defect, UiO-66-Cu^I^, and UiO-66-Cu^II^ exhibit NH_3_ uptakes of 11.8, 12.6, and 16.9 mmol g^–1^ (Figure 1a–c), respectively, comparable with state-of-the-art materials (Table S7). The isotherms display apparent hysteresis loops (Figure S3), indicating the presence of strong host–guest interactions at the binding sites within the two types of cages of the framework (tetrahedral and octahedral cages with diameters of 7 and 9 Å, respectively). UiO-66-defect, UiO-66-Cu^I^, and UiO-66-Cu^II^ show high packing density of NH_3_ of 0.52, 0.55, and 0.74 g cm^–3^, respectively, demonstrating efficient volumetric storage of NH_3_ (Table S3). It is worth noting that the packing density of UiO-66-Cu^II^ is comparable to that of liquid NH_3_ (0.68 g cm^–3^) at 240 K. All three MOFs show high stability toward pressure-swing NH_3_ adsorption with retention of the structure, porosity, and NH_3_ uptakes over at least 15 cycles (Figures 1e, S1, S4, and S6). This is in direct contrast to reported MOFs incorporating four- or five-coordinated open Cu(II) sites that lead to irreversible sorption of NH_3_ and structural degradation upon desorption.^19,28−30^ Upon desorption under pressure-swing conditions, higher residues of NH_3_ in UiO-66-Cu^I^ and UiO-66-Cu^II^ (49–67%) were observed compared with UiO-66-defect (27–30%), attributed to the interactions between NH_3_ molecules and Cu sites (*vide infra*). The residual NH_3_ in all three systems can be fully released *via* heating, reflecting a relatively strong binding of NH_3_ in these MOFs. The excellent ability of UiO-66-defect, UiO-66-Cu^I^, and UiO-66-Cu^II^ to capture NH_3_ at low concentrations (630 ppm) has been confirmed by dynamic breakthrough experiments at 298 K, where the dynamic NH_3_ uptakes were calculated to be 2.07, 3.07, and 4.15 mmol g^–1^, respectively (Figure 1d). The introduction of Cu(II) sites leads to 100% enhancement of the dynamic NH_3_ adsorption capacity at low concentrations, which is highly desirable for the capture of NH_3_ as a pollutant and/or at low concentrations. With increasing loading of NH_3_, the isosteric heat of adsorption (*Q*_st_) increases, and the adsorption entropy (Δ*S*) decreases for all three MOFs, indicating the presence of strong intermolecular interactions and ordering of adsorbed NH_3_ molecules in the pore (Figure S5). As expected, UiO-66-Cu^II^ shows higher *Q*_st_ than UiO-66-defect and UiO-66-Cu^I^ (up to 55, 40, and 35 kJ mol^–1^, respectively).

### Determination of the Binding Sites for Adsorbed ND_3_

**Figure 2:**
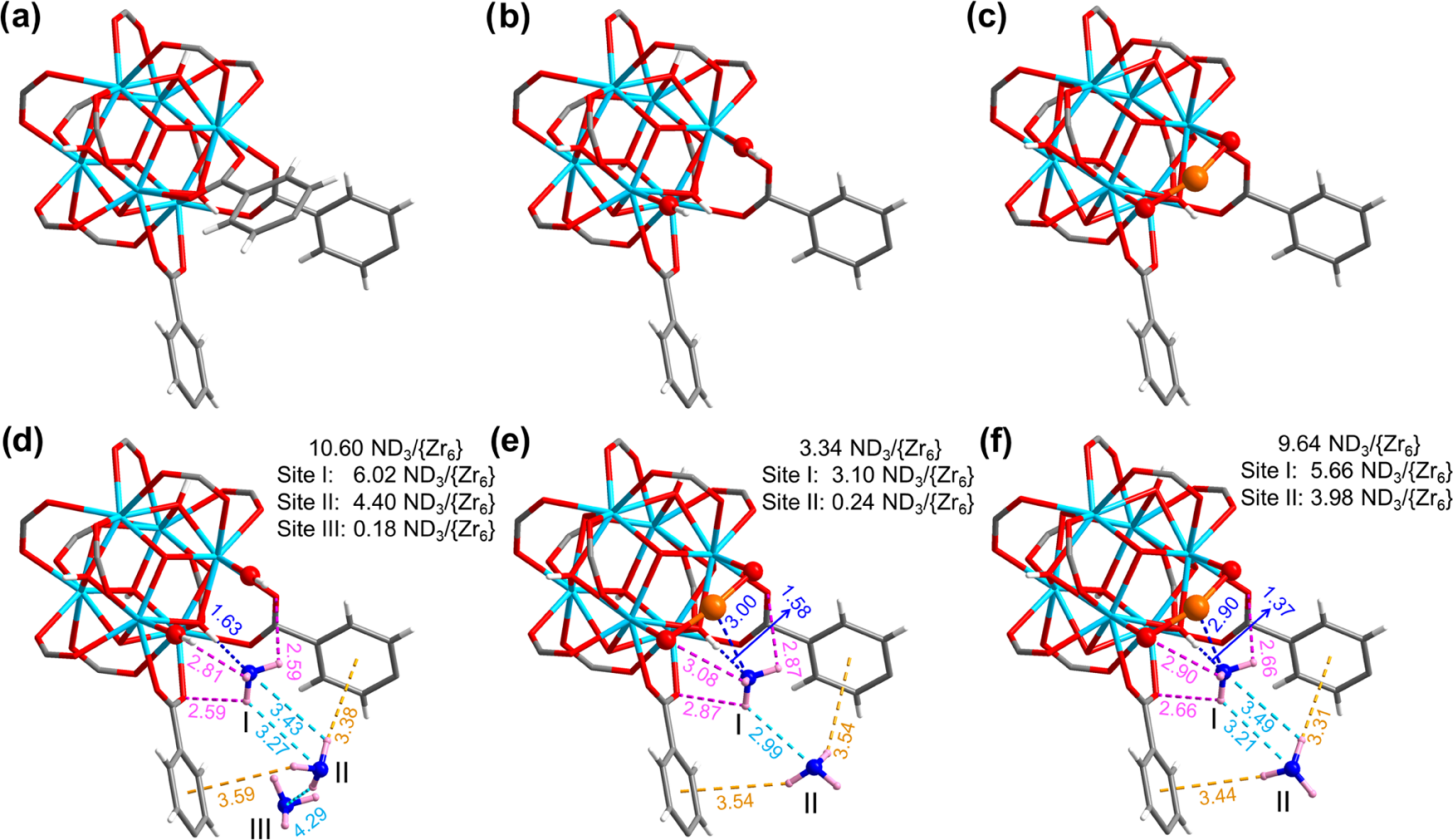
Structures of {Zr_6_} clusters in UiO-66 (a) without and (b) with a defect site in terms of a missing ligand and (c) in UiO-66-Cu^II^. Views of the binding sites of ND_3_ in (d) UiO-66-defect and UiO-66-Cu^II^ at (e) low and (f) high loadings, respectively. All structures were derived from Rietveld refinements of the NPD data collected at 7 K (C, gray; O, red; Zr, sky blue; Cu, orange; H, white; N, blue; D, pink).

Rietveld refinements of the *in situ* NPD data collected at 7 K illustrate the binding of ND_3_ to the −OH defect site in UiO-66-defect·10.6ND_3_ and to the atomically dispersed Cu(II) sites in UiO-66-Cu^II^·3.34ND_3_ and UiO-66-Cu^II^·9.64ND_3_. An additional low loading of ND_3_ was conducted for UiO-66-Cu^II^ to better elucidate the precise role of Cu(II) sites in binding ND_3_ at low concentrations. The structures of bare UiO-66 with and without a defect site are shown in Figures 2b and 2a, respectively. In UiO-66-defect·10.6ND_3_, three distinct binding sites (I, II, and III) are found (Figures 2d and S14). The primary binding site (Site I) of ND_3_ (occupancy of 6.02 ND_3_/{Zr_6_}) is anchored by two −OH groups at the defect site, forming a series of strong host–guest hydrogen bonds to the μ_3_-OH and defect −OH groups [O_μ3_–H···ND_3_ = 1.63(8) Å; ND_3_···O_defect_–H = 2.81(7) Å; and ND_3_···O_carboxylate_ = 2.59(1) Å]. Compared with the ND_3_@UiO-67,^21^ a stronger hydrogen bond between the defect −OH groups and ND_3_ molecules is observed in ND_3_@UiO-66-defect [O_μ3_–H···ND_3_ = 1.96(1) and 1.63(8) Å, respectively], resulting in higher NH_3_ uptake (8.4 and 11.8 mmol g^–1^, respectively). Site II (occupancy of 4.40 ND_3_/{Zr_6_}) is bound to the framework mainly *via* electrostatic interactions [ND_3_···aromatic rings = 3.38(12)–3.59(29) Å], further supplemented by intermolecular hydrogen bonding between adsorbed ND_3_ molecules [ND_3_^I^···ND_3_^II^ = 3.27(1)–3.43(32) Å]. Site III (occupancy of 0.18 ND_3_/{Zr_6_}) exhibits no direct interaction with the framework and is stabilized by the interaction with adjacent ND_3_ molecules at Site II through hydrogen bonding [ND_3_^II^···ND_3_^III^ = 4.29(13) Å].

**Figure 3:**
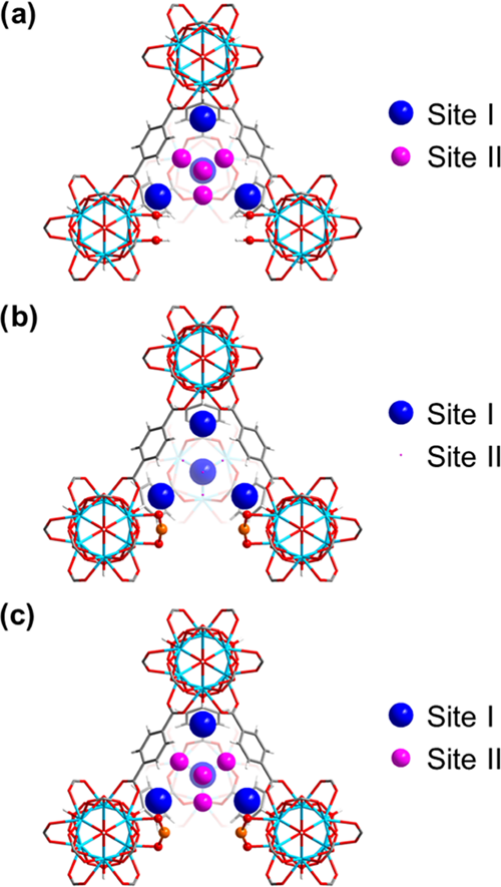
Distribution of adsorbed ND_3_ molecules within the tetrahedral cage in (a) UiO-66-defect·10.6ND_3_, (b) UiO-66-Cu^II^·3.34ND_3_, and (c) UiO-66-Cu^II^·9.64ND_3_ as determined from the refinement of NPD data. The radii of the colored balls of Site I (blue) and Site II (pink) are proportional to their crystallographic occupancies. (a) 6.02 ND_3_/{Zr_6_} for Site I and 4.40 ND_3_/{Zr_6_} for site II; (b) 3.10 ND_3_/{Zr_6_} for Site I and 0.24 ND_3_/{Zr_6_} for Site II; (c) 5.66 ND_3_/{Zr_6_} for Site I and 3.98 ND_3_/{Zr_6_} for Site II.

The Cu(II) ion in UiO-66-Cu^II^ binds to two oxygen centers and shows a near-linear coordination geometry (Figure 2c). The crystal structures for UiO-66-Cu^II^·3.34ND_3_ and UiO-66-Cu^II^·9.64ND_3_ determined by NPD both show two binding sites (Figures 2e,f and S15). Site I is anchored simultaneously by the Cu(II) site [Cu^II^···ND_3_ = 2.90(8)–3.00(6) Å] and adjacent hydroxyl groups *via* hydrogen bonding [O_μ3_–H···ND_3_ = 1.37(42)–1.58(1) Å; ND_3_···O_defect_ = 2.90(3)–3.08(2) Å; ND_3_···O_carboxylate_ = 2.66(3)–2.87(1) Å]. Site II is stabilized through electrostatic interactions [ND_3_···aromatic rings = 3.31(27)–3.54(39) Å], supplemented by intermolecular hydrogen bonding with surrounding ND_3_ molecules [ND_3_^I^···ND_3_^II^ = 2.99(3)–3.49(59) Å]. At higher ND_3_ loading, the occupancy of ND_3_ molecules at Site I increases by 83% from 3.10 to 5.66 ND_3_/{Zr_6_}. In contrast, the occupancy increases by *ca.* 16 times at Site II from 0.24 to 3.98 ND_3_/{Zr_6_} (Figure 3), unambiguously demonstrating the critical role of unique Cu(II) sites in binding ND_3_ at low concentrations, consistent with the ultrahigh dynamic uptake of NH_3_ at low concentrations. The overall binding distances decrease slightly upon increased loading, indicating stronger host–guest interactions between ND_3_ and the framework. Interestingly, hydrogen/deuterium (H/D) site-exchange^31,32^ is also observed between the μ_3_-OH group and adsorbed ND_3_ molecules in both UiO-66-defect and UiO-66-Cu^II^, suggesting the direct interaction *via* proton exchange between ND_3_ and μ_3_-OH. Cooperative {ND_3_}_∞_ networks are observed in both systems with intermolecular hydrogen bonds [ND_3_···ND_3_ = 2.99(3)–4.29(13) Å; Figures 3, S14, and S15]. This is reminiscent of the structure of condensed ND_3_ in solid state, and is consistent also with the observed increase in *Q*_st_, with increasing loading of NH_3_ between 3.0 and 6.7 mmol g^–1^. Importantly, this study represents the first example of structural elucidation of NH_3_ binding in MOFs containing open metal sites.

### Studies of Host–Guest Binding Dynamics

**Figure 4:**
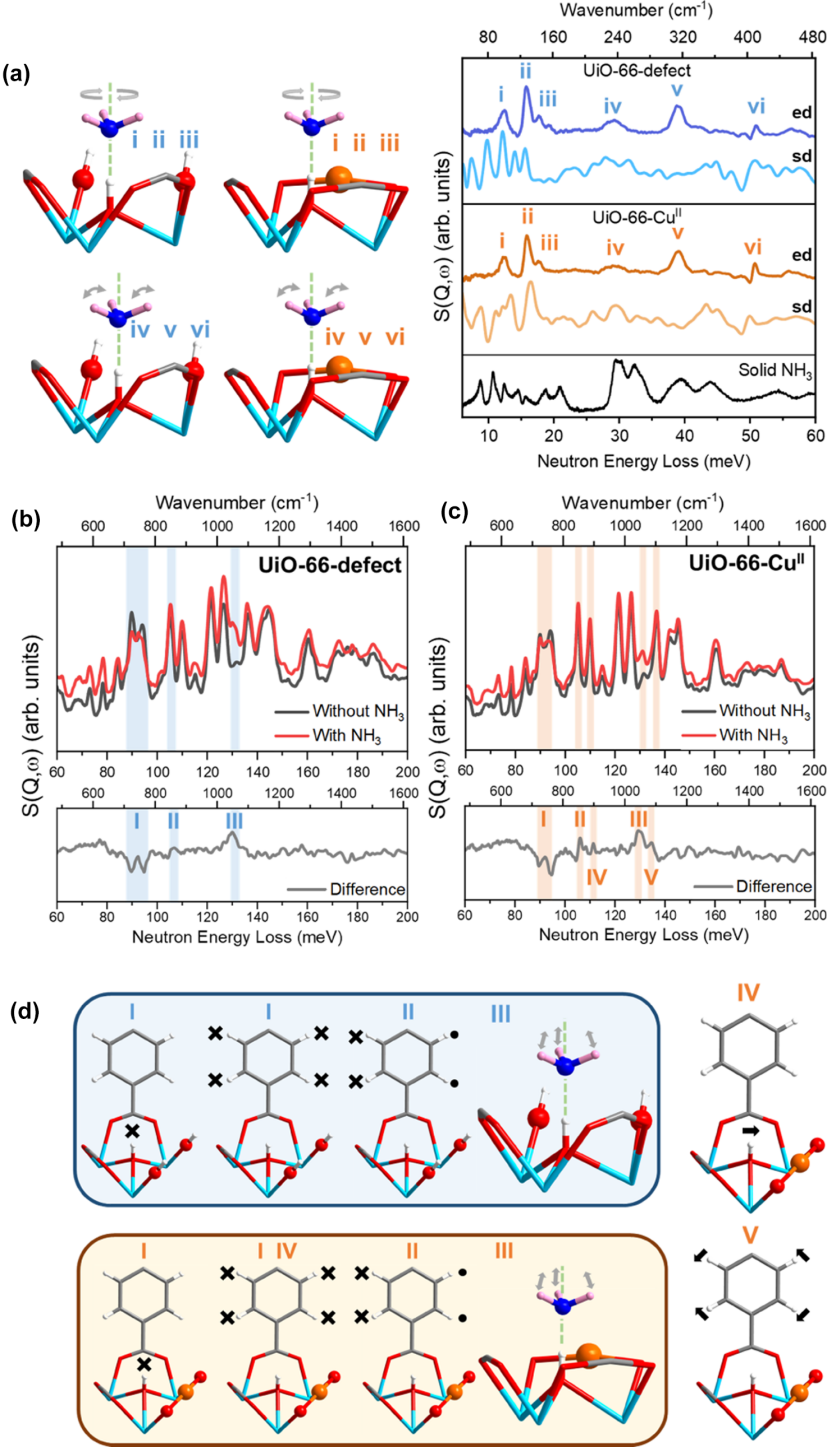
Views of *in situ* INS spectra, the DFT-calculated spectra, and the corresponding vibrational modes for UiO-66-defect and UiO-66-Cu^II^, before and after NH_3_ loading. Difference spectra were obtained by subtraction of the INS spectra of the bare MOF from that for the NH_3_-loaded MOF and are marked as ed (experimental difference spectra) and sd (simulated difference spectra). (a) Comparison of vibrational modes between solid NH_3_ at 7 K (8.7–21.0 meV translational modes; 29.4–32.3 meV librational modes; 39.3–54.4 meV rocking modes), and adsorbed NH_3_ in the MOF. (b, c) Experimental difference INS spectra for UiO-66-defect and UiO-66-Cu^II^ upon NH_3_ adsorption in the higher energy range. (d) Selected vibrational modes of UiO-66-defect and UiO-66-Cu^II^.

By combining *in situ* INS and DFT calculations, the vibrational modes of adsorbed NH_3_ molecules and that of the framework host can be deconvoluted and assigned to interpret the rapid dynamics of the system. The experimental and simulated INS spectra showed excellent agreement for bare and NH_3_-loaded UiO-66-defect and bare and NH_3_-loaded UiO-66-Cu^II^. By subtracting the spectra of the MOF and sample cell from the NH_3_-loaded samples, difference INS spectra can be obtained. For the loading of 2NH_3_ per {Zr_6_} cluster, the NH_3_ molecules are primarily adsorbed at Site I (Figure 4a). In UiO-66-defect and UiO-66-Cu^II^, librational modes of adsorbed NH_3_ molecules around its *C*_3_ axis are observed at 15.8 and 17.7 meV. The peaks at 29.3, 38.8, and 51.0 meV in NH_3_-loaded UiO-66-defect (29.4, 39.1, and 50.7 meV in UiO-66-Cu^II^) correspond to the rocking motions of NH_3_ around the N center. The significant red shift of these peaks compared to solid NH_3_ (librational modes at 29.4–32.3 meV; rocking modes at 39.3–54.4 meV) are attributed to the rotational flexibility of NH_3_ in its adsorbed local environment, in contrast to the NH_3_ molecules in the solid state connected by the three-dimensional hydrogen-bonding network. The peaks (or dips) in the experimental difference INS spectra in the high energy region, mostly corresponding to variations of system dynamics upon NH_3_ binding, are also assigned (Figure 4b–d). Three changes are observed in both UiO-66-defect and UiO-66-Cu^II^ systems on loading with NH_3_ corresponding to the following vibrational modes: (I) at 92.3 meV, the μ_3_-OH in Zr–O–H plane bending and H–C out-C_6_-plane deformation; (II) at 105 meV, the H–C out-C_6_-plane deformation; (III) at 130 meV, NH_3_ umbrella motion. For UiO-66-Cu^II^, two extra changes are observed in the difference spectrum and they correspond to (IV) at 110 meV, the μ_3_-OH out of Zr–O–H plane bending and H–C out-C_6_-plane deformation, and (V) at 137 meV, the H–C in-C_6_-plane bending. The introduction of Cu(II) sites to the structural defects contributes to an increase of the acidity at the μ_3_-OH moiety with stronger μ_3_-OH out-of-plane (Zr–O–H) bending in UiO-66-Cu^II^ upon NH_3_ adsorption, as evidenced by peak IV. This is in excellent agreement with the shorter distance of O–H···ND_3_ hydrogen bonds observed in the NPD study [O_μ3_-H···ND_3_ of 1.58(1) and 1.63(8) Å in UiO-66-Cu^II^ and UiO-66-defect, respectively]. Compared with UiO-66-defect, a slight increase in the intensity of the H–C in-C_6_-plane bending peak is observed in UiO-66-Cu^II^ (peak V), which could be related to the shorter distance between NH_3_ and the aromatic ring in this system [ND_3_···aromatic ring of 3.49(1) Å in UiO-66-Cu^II^ and 3.53(1) Å in UiO-66-defect]. Overall, the INS/DFT results afford excellent agreement with the structural models derived from NPD data and new insights into the binding dynamics of NH_3_ in these decorated MOFs.

### Investigation of the Host–Guest Interactions

**Figure 5:**
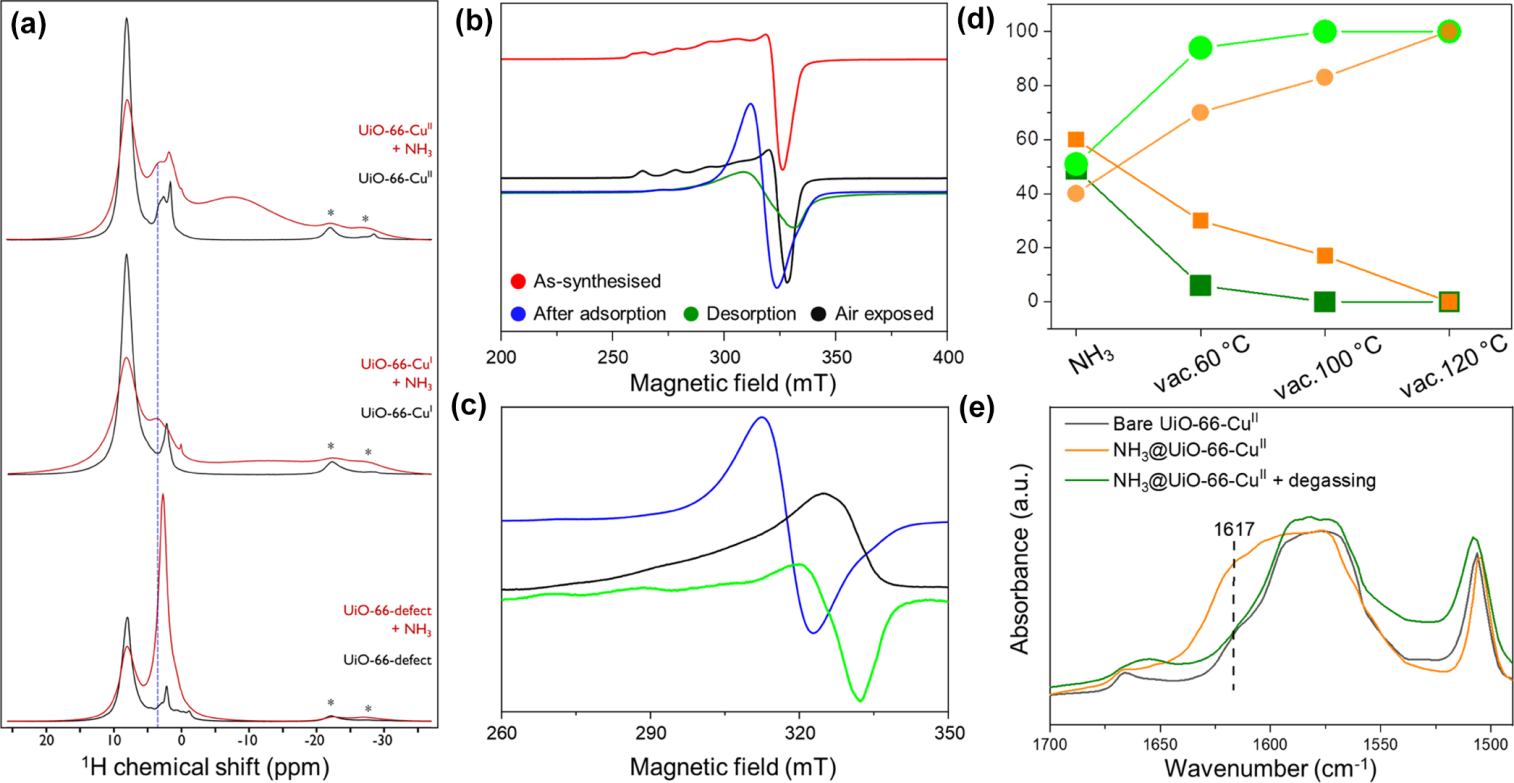
(a) ^1^H DEPTH MAS NMR spectra of bare (black) and NH_3_-loaded (red) UiO-66-defect (bottom), UiO-66-Cu^I^ (middle), and UiO-66-Cu^II^ (top). The spectra were recorded at 9.4 T using a MAS frequency of 12 kHz. The dashed vertical blue line highlights the signal from pore-confined NH_3_ in the UiO-66-Cu^I^ and UiO-66-Cu^II^ samples, and the asterisks denote the position of spinning sidebands. (b) X-band (9.4 GHz) EPR spectra of UiO-66-Cu^II^ recorded at 40 K before adsorption of NH_3_ (red, pre-activated solvated form), after adsorption of NH_3_ (blue), desorption of NH_3_ (green), and after exposure to the air for more than 24 h (black). (c) X-band (9.4 GHz) EPR spectra of UiO-66-Cu^II^ at 6 K after NH_3_ loading. Blue: CW spectra; black: echo-detected spectra recorded with π/2 = 16 ns and τ = 150 ns; and light green: derivative of echo-detected spectra recorded with π/2 = 16 ns and τ = 150 ns. (d) Relative quantities of the broad (square, orange and deep green) and isolated Cu(II) (circle, light orange and lime green) EPR signals upon degassing NH_3_@UiO-66-Cu^II^ (orange) and NH_3_@UiO-66-Cu^I^ (green) with heating under dynamic vacuum (see SI for details). (e) *In situ* infrared spectra of UiO-66-Cu^II^ upon adsorption and desorption of NH_3_.

*In situ* infrared and ssNMR experiments were carried out to further investigate the interactions of NH_3_ in these porous materials. Upon introduction of NH_3_, depletion of the O–H stretching bands at 3673 and 3646 cm^–1^ was observed (Figure S16), consistent with the binding of NH_3_ molecules to μ_3_-OH and defect −OH sites. Interestingly, an additional band was observed at 1617 cm^–1^ for NH_3_-loaded UiO-66-Cu^II^ (Figure 5e) assigned to asymmetric vibration of adsorbed NH_3_ molecules on Lewis acid sites [Cu(II) in this case].^33−35^ The presence of possible charge transfer between Cu(II) sites and bound NH_3_ molecules has been confirmed by *in situ* UV–vis spectra, which show an additional broad absorption band centered at around 680 nm in NH_3_-loaded UiO-66-Cu^II^ (Figure S17).^36^ The presence of strong binding of NH_3_ to Cu(I) and Cu(II) sites was also confirmed by temperature-programmed desorption of NH_3_ (NH_3_–TPD) (Figure S18) and ^1^H ssNMR spectroscopy (Figure 5a). The additional TPD peaks at higher temperatures (150–300 °C, Figure S18b) for UiO-66-Cu^I^ and UiO-66-Cu^II^, which are not observed in UiO-66-defect, indicate stronger binding of NH_3_ at these Cu sites. In addition, the desorption peaks for UiO-66-Cu^II^ appear at higher temperatures compared with those of UiO-66-Cu^I^, suggesting a stronger {Cu(II)···NH_3_} interaction than {Cu(I)···NH_3_}, consistent with the adsorption and breakthrough results. These conclusions are supported further by the corresponding ^1^H magic angle spinning (MAS) NMR spectra of the NH_3_-loaded materials (Figure 5a). For UiO-66-defect, a large narrow signal from NH_3_ is observed (FWHM ∼ 650 Hz, centered at δ{^1^H} = 2.8 ppm), suggesting rapid relative motion of NH_3_ in the pores. For UiO-66-Cu^II^ and UiO-66-Cu^I^, this large peak is absent but a broad signal (FWHM ∼ 2 kHz, centered at δ{^1^H} = 3.7 ppm) is present that stems from pore-confined NH_3_ (Figure S19). Furthermore, for UiO-66-Cu^I^ and UiO-66-Cu^II^, very broad signals are observed at negative chemical shifts (FWHM ∼ 7 kHz, centered at δ{^1^H} = −7.6 ppm for UiO-66-Cu^II^ and δ{^1^H} = −15.0 ppm for UiO-66-Cu^I^) corresponding to metal-bound NH_3_,^37^ again consistent with the infrared and UV–vis spectroscopic studies. One-dimensional (1D) ^13^C and 2D ^1^H–^13^C dipolar correlation MAS NMR spectra (Figure S19) also indicate a hydrogen-bonding interaction between NH_3_ molecules and the carboxylate moieties from the organic linkers of the MOFs.

The strong interaction between NH_3_ and Cu(II) site was further elucidated by EPR spectroscopy. UiO-66-Cu^II^ in its hydrated form has an X-band continuous wave (CW) EPR spectrum (Figure 5b), with axial (within the resolution of the experiment) spin Hamiltonian parameters [*g*_*x*,*y*_ = 2.074, *g_z_* = 2.320, *A*_*z*(Cu)_ = 480 MHz], typical of isolated Cu(II) ions with a d_*x*^2^–*y*^2^_ or d*_xy_* ground state and consistent with water coordinated in the *xy* plane; the latter is confirmed by HYSCORE measurements.^22^ Dehydration of the sample leads to loss of the coordinated water (Figures S20, S21, and S23) and significant intensity loss in the CW EPR spectrum.^22^ This phenomenon has been observed in several Cu(II)-doped zeolites [without reduction to Cu(I)], attributed to unusual low-coordinate geometries that can lead to near-degenerate ground states.^38^ This is also consistent with the NPD model that suggests a pseudo-linear geometry at the Cu(II) site (Figure 2c) and with the observation that the spectra (CW EPR, HYSCORE) of the solvated system are not restored by exposure to dry O_2_ but are restored by exposure to air *via* the uptake of moisture.

Adsorption of NH_3_ in an activated sample of UiO-66-Cu^II^ led to the recovery of the intensity of the signal within the CW EPR spectrum, indicating that the adsorbed NH_3_ interacts with the Cu(II) sites. Two components are observed: (i) an isolated Cu(II) signal with modified parameters [*g_x,y_* = 2.07, *g_z_* = 2.27, *A*_*z*(Cu)_ = 530 MHz] and (ii) an unresolved, broad signal at *g* ≈ 2.115 (Figures 5b and S20, and Table S5). Only the former is observed in echo-detected EPR spectroscopy (Figures 5c and S22), demonstrating their different origin and that the species giving rise to the broad signal relaxes quickly. The observed decrease in *g_z_* and increase in *A*_*z*(Cu)_ (compared to the hydrated form) of the anisotropic component are consistent with a mixed O/N-donor set, and similar changes have been observed in NH_3_ binding in Cu-doped zeolites.^39^ The origin of the broad signal is less clear: the lack of resolution and rapid relaxation may indicate exchange interactions between the Cu(II) ions. The nearest possible intra- and inter-node Cu···Cu distances are 4.4 and 5.8 Å, respectively, and an interaction would only need to be a few hundred MHz to affect the CW EPR response significantly. This could be mediated by the hydrogen-bonding network of adsorbed NH_3_ molecules within the tetrahedral cages. Since the defects will be distributed statistically, both coupled and uncoupled spectra could be observed. An alternative explanation for the broad signal would be a fluxional process, which averages the EPR response. However, the spectra are unchanged on cooling to 4 K, which should freeze out any such process. Similar broad, near-isotropic CW EPR signals have been observed on NH_3_ loading on the Cu(II)-MOF, HKUST-1, tentatively attributed to spin-exchange phenomena.^29^ In contrast to the HKUST-1 study, where the changes in NH_3_ adsorption were irreversible,^29^ the CW EPR and HYSCORE spectra of UiO-66-Cu^II^ can be readily regenerated by degassing and exposure to air, confirming excellent stability and reversibility of this system (Figures 5b, S21, and S23). At 10^–7^ mbar at different temperatures, the broad signal is lost first (Figure S20), consistent with preferential loss of NH_3_ molecules at Site II/III, which disrupts the hydrogen-bonding network that facilitates the Cu···Cu interaction.

Similar EPR spectra and behavior are found for UiO-66-Cu^I^, which indicates the presence of a minor amount of Cu(II) ions along with Cu(I) sites after the reduction. To compare the strength of Cu···NH_3_ binding in UiO-66-Cu^II^ and UiO-66-Cu^I^ materials, the desorption and regeneration processes after NH_3_ adsorption were compared (Figures 5d and S21). The broad isotropic signal is lost more quickly for UiO-66-Cu^I^, demonstrating stronger binding in the UiO-66-Cu^II^ system, consistent with the TPD and ssNMR analyses and the isothermal adsorption and breakthrough results.

## Conclusions

In summary, robust UiO-66 materials incorporating atomically dispersed defects and open Cu(I) and Cu(II) sites show high and reversible NH_3_ adsorption capacities. While the decoration of defects with open Cu(I) and Cu(II) sites exhibits little change to the BET surface area (1111–1135 m^2^ g^–1^), the latter Cu(II) system shows 43 and 100% enhancements in the static and dynamic adsorption of NH_3_, respectively, compared with UiO-66-defect. This places UiO-66-Cu^II^ as one of the state-of-the-art NH_3_ sorbents. The host–guest interactions between the frameworks and adsorbed NH_3_ molecules have been investigated comprehensively at the molecular level. *In situ* NPD, ssNMR, EPR, IR, UV–vis, and INS/DFT studies have established the binding interactions between NH_3_ and defect sites and the critical role of low-coordinate Cu(II) sites in stabilizing NH_3_ molecules has been determined unambiguously. This is distinct from four and five-coordinated Cu(II) sites that lead to irreversible structural degradation upon desorption of NH_3_. By combining NH_3_–TPD, *in situ* ssNMR, IR, UV–vis, and EPR experiments, the host–guest interactions have been revealed, and this is accompanied by a reversible change of the unique, near-linear coordination geometry of Cu(II) sites as a function of NH_3_ binding. This is the structural origin of the observed reversible NH_3_ adsorption in this system involving open metal sites. These findings showcase the designed tuning of active sites in MOFs that can result in top-performing NH_3_ adsorption without altering the porosity of a given material. We anticipate that this study will provide key insights into the design and preparation of new efficient sorbents for NH_3_*via* the full control of active sites with atomic precision.

## Supporting Information Available

Additional experimental details, adsorption isotherms, breakthrough experiments, views of crystal structures, XAFS, powder X-ray diffraction (PXRD), NPD, IR, UV–vis, NH_3_–TPD, EPR, and ssNMR data (Figures S1–S23 and Tables S1–S11) (PDF)

## Accession Codes

CCDC 2124989–2124993 contain the supplementary crystallographic data for this paper. These data can be obtained free of charge via www.ccdc.cam.ac.uk/data_request/cif, or by emailing data_request@ccdc.cam.ac.uk, or by contacting The Cambridge Crystallographic Data Centre, 12 Union Road, Cambridge CB2 1EZ, UK; fax: +44 1223 336033.
