# Supplementary material for: Direct Observation of Ammonia Storage in UiO-66 Incorporating Cu(II) Binding Sites
Source: J Am Chem Soc. 2022 May 9;144(19):8624–32. doi: 10.1021/jacs.2c00952 (PMC9121371; doi:10.1021/jacs.2c00952)
Supplement: Supplementary file 1 — ja2c00952_si_001.pdf [file ja2c00952_si_001.pdf]

Supporting Information

Direct observation of ammonia storage in UiO-66 incorporating Cu(II) binding sites

Yujie Ma,[1†] Wanpeng Lu,[1†] Xue Han,[1] Yinlin Chen,[1] Ivan da Silva,[2] Daniel Lee,[3] Alena M. Sheveleva,[1,4] Zi Wang,[1] Jiangnan Li,[1] Weiyao Li,[1] Mengtian Fan,[1] Shaojun Xu,[3,7,8] Floriana Tuna,[1,4] Eric J. L. McInnes,[1,4] Yongqiang Cheng,[5] Svemir Rudić,[2] Pascal Manuel,[2] Mark D. Frogley,[6] Anibal J. Ramirez-Cuesta,[5] Martin Schröder[1]* and Sihai Yang[1]*

1. Department of Chemistry, University of Manchester, Manchester, M13 9PL, UK
2. ISIS Facility, Science and Technology Facilities Council, Rutherford Appleton Laboratory, Chilton, OX11 0QX, UK
3. Department of Chemical Engineering and Analytical Science, University of Manchester, Manchester M13 9PL, UK
4. Photon Science Institute, University of Manchester, Manchester, M13 9PL, UK
5. Neutron Scattering Division, Neutron Sciences Directorate, Oak Ridge National Laboratory, Oak Ridge, Tennessee 37831, USA
6. Diamond Light Source, Harwell Science Campus, Oxfordshire, OX11 0DE, UK
7. UK Catalysis Hub, Research Complex at Harwell, Rutherford Appleton Laboratory, Harwell OX11 0FA, UK
8. School of Chemistry, Cardiff University, Cardiff, CF10 3AT, UK

†: These authors contributed equally to this work.

List of Contents

1. Experimental Section

  1.1 Synthesis of Materials

  1.2 Characterisation of Materials

  1.3 Gas Adsorption Isotherms and Breakthrough Experiments

  1.4 Temperature-Programmed Desorption of Ammonia (NH_3-TPD)

2. Adsorption Isotherms, Cycling Experiments and Calculation of Q_{st}

3. Neutron Powder Diffraction (NPD) Patterns

4. Additional Views of Crystal Structures

5. Infrared and UV-vis Spectroscopy

6. Solid-State Nuclear Magnetic Resonance (ssNMR) Spectroscopy

7. Electron Paramagnetic Resonance (EPR) Spectroscopy

8. Comparison of Isothermal and Dynamic Ammonia Adsorption Capacity in MOFs

1. Experimental Section

1.1 Synthesis of Materials

All the reagents were used as received from commercial suppliers without further purification. Synthesis of UiO-66-defect and UiO-66-Cu^{I} and UiO-66-Cu^{II} was carried out using our previously reported methods.[1,2]

Synthesis of UiO-66-defect. Terephthalic acid (125 mg) and zirconium tetrachloride (167 mg) were dissolved in a mixture solution containing 50 mL of N,N-dimethylformamide (DMF) and 3.5 mL of formic acid. The mixture was transferred into a teflon lined bomb, sealed and heated at 120 °C for 24 h. The product was collected by centrifugation (10,000 rpm, 3 min) and washed with DMF and acetone. The sample of UiO-66-defect was dried under dynamic vacuum before further use.

Synthesis of UiO-66-Cu^{II} and UiO-66-Cu^{I}. To prepare UiO-66-Cu^{II}, 500 mg of UiO-66-defect was added to a solution containing $CuCl_2$ (500 mg) dissolved in DMF (10 mL). The mixture was sonicated for 1 min and then heated at 85 °C for 24 h. The product was collected by centrifugation (10,000 rpm, 3 min) and washed with DMF and acetone. UiO-66-Cu^{II} was dried under dynamic vacuum before further use. UiO-66-Cu^{II} was reduced in a tube furnace with 5% H_2 flow (diluted in Ar) at 250 °C for 2 h to obtain UiO-66-Cu^{I}. The loadings of Cu in UiO-66-Cu^{II} and UiO-66-Cu^{I} are 2.62% and 2.71%, respectively, as determined by inductively coupled plasma-optical emission spectrometry (ICP-OES).

1.2 Characterisation of Materials

Powder X-ray diffraction (PXRD) patterns were collected using a Philips X'pert X-ray diffractometer (40kV and 30 mA) using Cu-Kα radiation (λ = 1.5406 Å). N_2 adsorption isotherms were measured on a Tristar II PLUS (Micrometrics) instrument at 77 K. DRIFTS experiments were carried out on Shimadzu IRAffinity-1S IR Spectrometer with a Praying Mantis attachment manufactured by Harrick. Ultraviolet-visible (UV-vis) spectra were recorded on a UV-vis spectrophotometer (Shimadzu, UV 2600). ICP-OES analysis was performed on a Thermo Scientific iCAP 6300 Duo. *Ex situ* X-ray absorption spectroscopy (XAS) measurements were performed at the Cu K-edge (8979 eV) on the B18 beamline at the Diamond Light Source, Didcot, UK. Measurements were performed in transmission mode using a QEXAFS setup with fast-scanning Si(111) double crystal monochromators for the Cu edge. The data processing was performed using IFEFFIT with the Demeter package (Athena and Artemis). Fitting parameters: $S_0^2 = 0.86$; fit range $33 < k(Å-2) < 12.4$, $1 < R(Å) < 3$; number of independent points = 11.7.

In situ gas-loaded synchrotron FTIR micro-spectroscopy was carried out at the Multimode InfraRed Imaging and Microspectroscopy (MIRIAM) beamline at the Diamond Light Source, Harwell Science Campus (UK). The instrument is comprised of a Bruker Hyperion 3000 microscope in transmission mode with 15× objective and condenser lenses and liquid N_2 cooled MCT detector, coupled to a Bruker Vertex 80V Fourier Transform IR interferometer using radiation generated from a bending magnet source. Spectra were collected (512 scans) in the range 4000–400 cm^{-1} at 4 cm^{-1} resolution with an infrared spot size at the sample of approximately 30 × 30 μm. Samples were placed onto a zinc selenide (ZnSe) disk and placed within a Linkam FTIR 600 gas-tight sample cell equipped with ZnSe windows, a heating stage and gas inlet and outlets. NH_3

was dosed volumetrically into the sample cell using mass flow controllers, the total flow rate being maintained at 100 mL min^{-1} for all experiments. The gases were directly vented to an exhaust system and the total pressure in the cell was maintained at 1 bar for all experiments. Before NH_3 adsorption, the MOF sample was desolvated under a flow of dry N_2 at 100 mL min^{-1} and 393 K for 2 h, and then cooled to room temperature under a continuous flow of N_2. For all MOF samples, the initial gas flow was pure N_2 at a flow rate 100 mL min^{-1}, which was then changed to 5 mL min^{-1} of NH_3 and 95 mL min^{-1} of N_2 (5% NH_3 in N_2). The sample was stabilized for 4 minutes under NH_3 flow. The spectrum of the regenerated/degassed MOF sample was acquired after the NH_3 was removed under a flow of dry N_2 at 100 mL min^{-1} at 298 K for 10 minutes and dry N_2 at 100 mL min^{-1} at 393 K for 30 mins.

Density functional theory (DFT) simulations were performed using CP2K (http://www.cp2k.org)[3] based on the mixed Gaussian and plane-wave scheme[4] and the Quickstep module.[5] The calculation used molecularly optimized Double-Zeta-Valence plus Polarization (DZVP) basis set,[6] Goedecker-Teter-Hutter pseudopotentials,[7] and the Perdew-Burke-Ernzerhof (PBE) exchange correlation functional.[8] The plane-wave energy cutoff was 400 Ry, and a DFT-D3 level correction for dispersion interactions, as implemented by Grimme *et al*,[9] was applied with a cutoff distance of 15 Å. The calculation was performed on Gamma point only with no symmetry constraint. Structural optimization was performed using the Broyden-Fletcher-Goldfarb-Shannon (BFGS) optimizer until the maximum force fell below 0.00045 Ry/Bohr (0.011 eV/Å). The finite displacement method was used for the phonon calculation, with incremental displacement of 0.01 Bohr (0.0053 Å). INS spectra were simulated using the OClimax software.[10]

For electron paramagnetic resonance (EPR) studies, the tube size and tube position in the cavity were kept constant. Desolvated UiO-66-Cu^{I} was prepared by a rapid transfer of sample after H_2 reduction from tube furnance into a J. Young X-band EPR tube (4 mm). The sample was purged with Ar for 2 h and evacuated at 10^{-7} mbar for 2 h at room temperature. Desolvated UiO-66-Cu^{II} was prepared by heating the sample at 120 °C in a J. Young X-band EPR tube at 10^{-7} mbar. For NH_3 adsorption studies, the sample in a J. Young X-band EPR tube was dosed with NH_3, and samples after dosing of NH_3 were evacuated under 10^{-7} dynamic vacuum at different temperatures (60/100/120 °C).

EPR echo-detected (ED) spectra were measured at X-band using a Hahn echo sequence, $\pi/2 - \tau - \pi - \tau$ – echo, with $\pi/2$ and π pulse lengths of 16 and 32 ns, respectively, and an inter-pulse delay τ of 150 ns. HYSCORE[11] spectra were measured at X-band using standard pulse sequence ($\pi/2 - \tau - \pi/2 - T1 - \pi - T2 - \pi/2 - \tau$ – echo); the length of mw pulses was $\pi/2$ = 16 ns and π = 26 ns. The time delay between first two pulses was taken as τ = 140 ns and τ = 200 ns, and starting values of T1 and T2 incrementing times were 100 ns. The (128x128) HYSCORE data array was recorded with a time increment of 16 ns, and then two-dimensional Fourier transform (FT) magnitude spectra were calculated.

Simulation of the EPR spectra was performed with the EasySpin/MATLAB toolbox, which employs the exact diagonalization of the spin Hamiltonian matrix.[12] The difference between the two Cu isotopes (^{63}Cu and ^{65}Cu) is included in the simulation program but the effect is not resolved at the linewidths observed.

1.3 Gas Adsorption Isotherms and Breakthrough Experiments

***Q_{st}* Calculation.** The isosteric enthalpy of adsorption (ΔH_n below) and entropies (ΔS_n) for NH_3 uptake were calculated as a function of loading (n), with all isotherms (273 K to 313 K) fitted to the van't Hoff isochore:

$$\ln(p)_n = \frac{\Delta H_n}{RT} - \frac{\Delta S_n}{R}$$

A plot of ln*(p)* versus $1/T$ at constant loading allows the differential enthalpy and entropy of adsorption and the isosteric enthalpy of adsorption (Q_{st}, n) to be determined.

Breakthrough Experiments. The MOF samples were activated at 393 K under dynamic vacuum for 16 h before breakthrough experiments, and 100 mg of desolvated sample was packed into fixed-bed reactors. The sample was then heated at 423 K under He flow for 2 h to achieve further activation. The fixed-bed was cooled to 298 K and the breakthrough experiment performed with a stream of 630 ppm NH_3 diluted in He. The flow rate of the gas mixture was 100 mL min^{-1}. A Matrix MG5 FTIR spectrometer was used to analyze the outlet gases for detection of NH_3, H_2O and CO_2. The gas concentration, C, of NH_3 at the outlet was compared with the corresponding inlet concentration C_0, where $C/C_0 = 1$ indicates complete breakthrough.[13]

1.4 Temperature-Programmed Desorption of Ammonia (NH_3-TPD)

NH_3-TPD experiments were carried out to test NH_3 binding on MOF materials. Typically, a stream of NH_3 diluted in He was passed through the activated sample. When the outlet concentration of NH_3 was equal to that of inlet, 100 mL min^{-1} of pure He was used to flush the sample for about 2 h, removing the surplus NH_3. The NH_3-TPD experiments were undertaken using a heating rate of 5 °C min^{-1} from 30 °C to 300 °C.

2. Adsorption Isotherms, Cycling Experiments and Calculation of Q_{st}

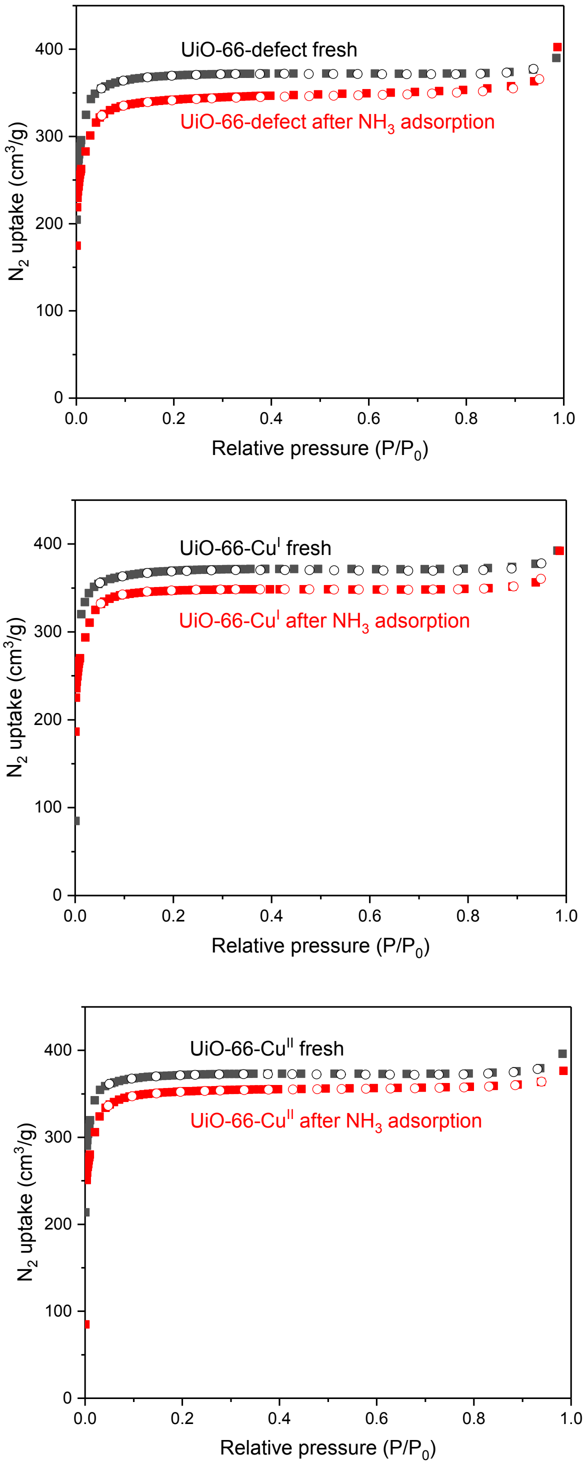

Figure S1. Nitrogen adsorption isotherms of UiO-66-defect, UiO-66-Cu^{I}, and UiO-66-Cu^{II} before (black) and after (red) NH_3 adsorption; top to bottom: UiO-66-defect, UiO-66-Cu^{I} and UiO-66-Cu^{II}.

Table S1. Summary of BET surface areas of the MOF materials before and after NH_3 adsorption (Calculated BET surface area, m^2 g^{-1}).

	UiO-66-defect	UiO-66-Cu^I	UiO-66-Cu^{II}
before NH_3 adsorption	1135	1111	1124
after NH_3 adsorption	1032	1049	1069

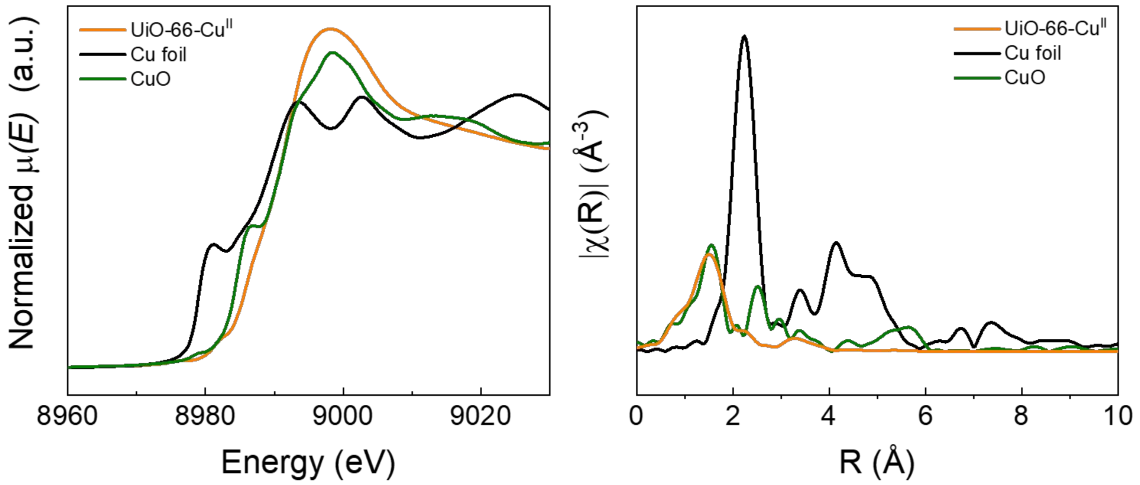

Figure S2. Normalized Cu K-edge XANES spectra of UiO-66-Cu^{II} and standard XANES spectra of Cu foil and CuO recorded as references (left). Plot of non-phase corrected Fourier transformed Cu K-edge EXAFS data for UiO-66-Cu^{II}, shown against Cu foil and CuO standard references (right).

Table S2. Cu K-edge EXAFS fitting parameters of UiO-66-Cu^{II}.

Sample	Absorber - Scatterer	R (Å)	σ^2 ($Å^2$)	E_0 (eV)	R_{factor}
UiO-66-Cu^{II}	Cu-O	1.95(1)	0.0073(1)	-4.7(1)	0.022

Note: Fitting parameters: $S_0^2 = 0.86$; fit range $3 < k(Å^{-2}) < 12.4$, $1 < R(Å) < 3$; number of independent points = 11.7.

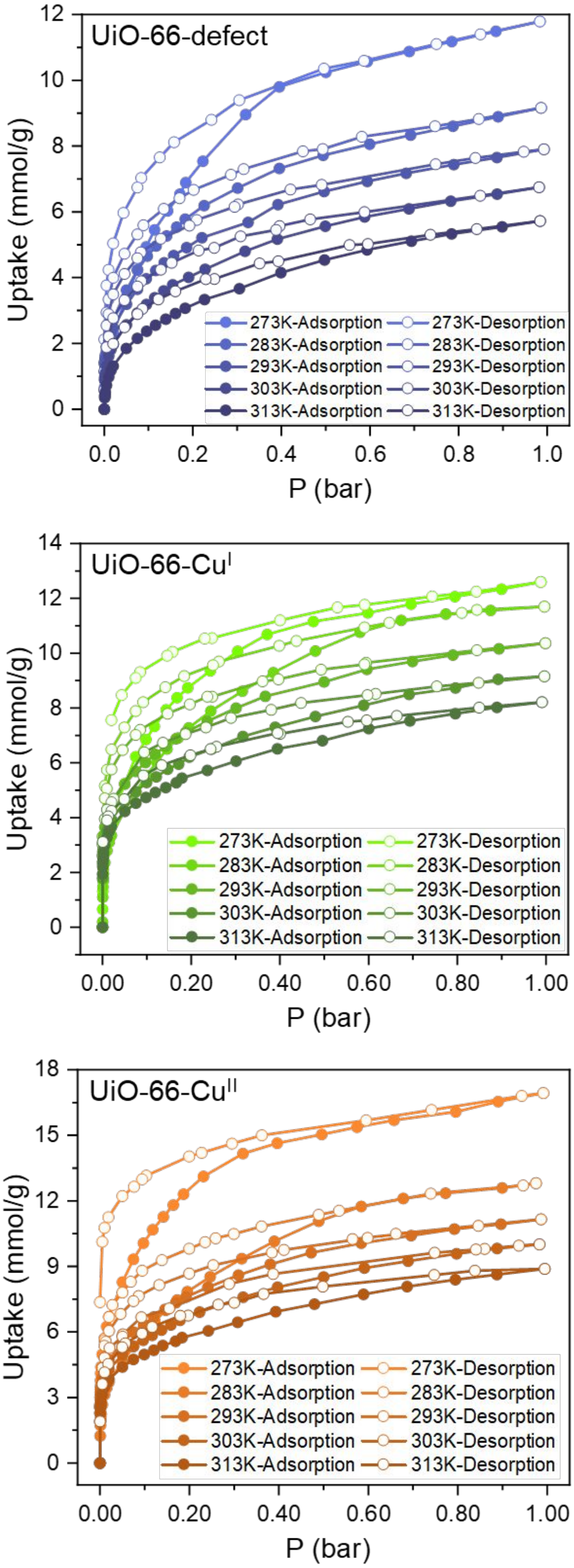

Figure S3. Adsorption-desorption isotherms of NH_3 between 273K and 313 K; top to bottom: UiO-66-defect, UiO-66-Cu^{I} and UiO-66-Cu^{II}).

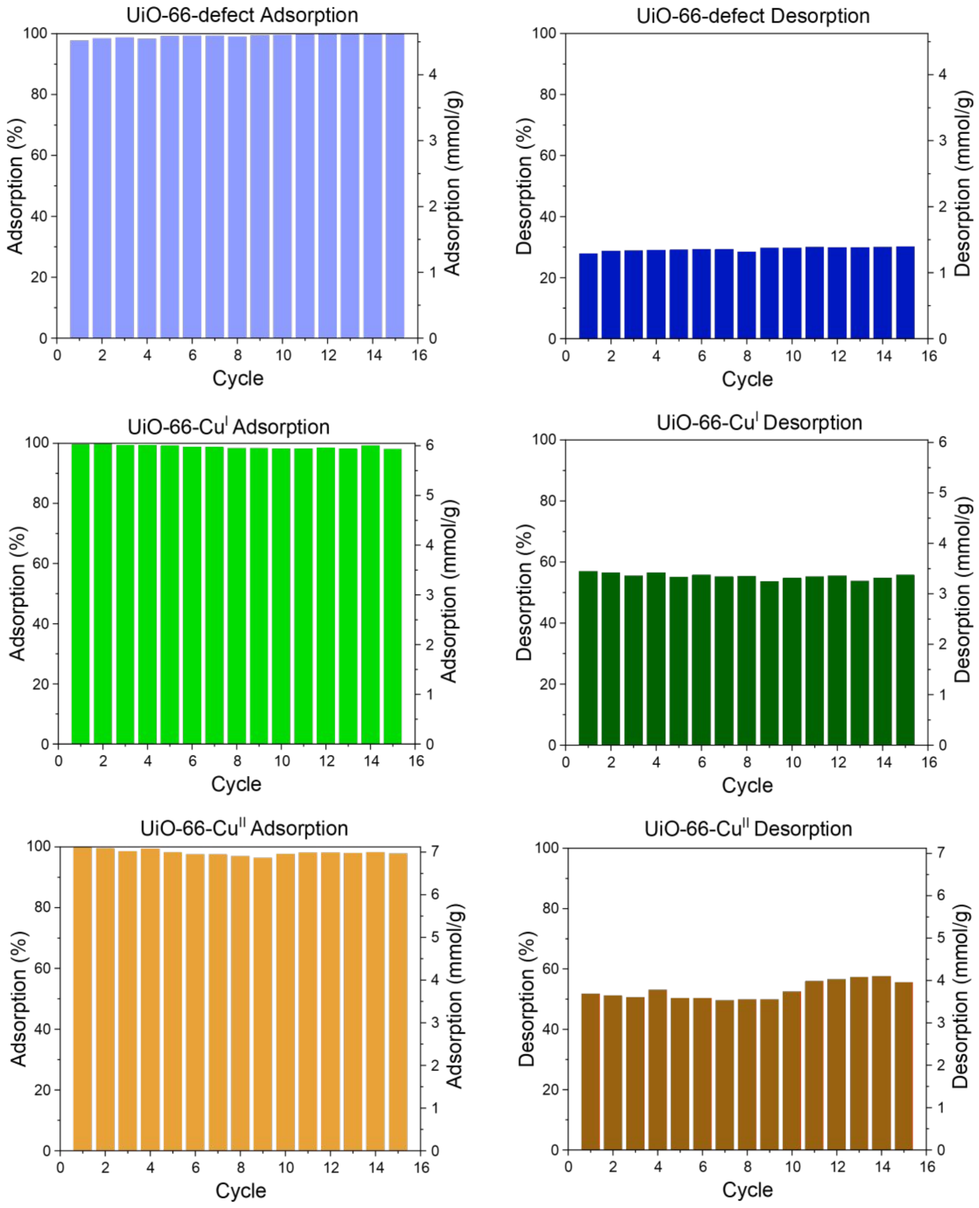

Figure S4. Cyclic adsorption-desorption of NH_3 at 298K between 0 and 0.15 bar; top to bottom: UiO-66-defect, UiO-66-Cu^I and UiO-66-Cu^{II}.

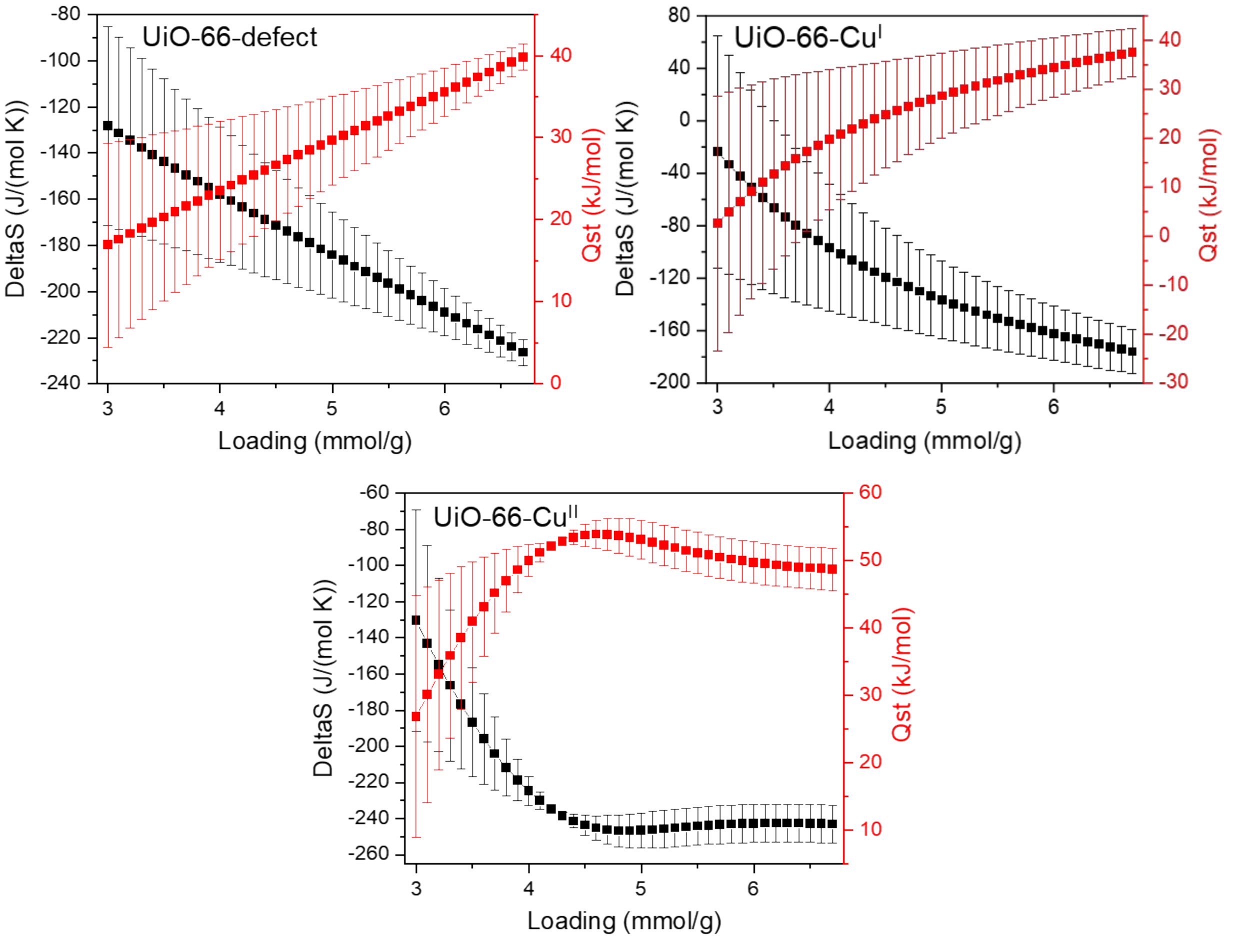

Figure S5. Isosteric enthalpy and entropy of adsorption at different NH_3 loadings; top to bottom: UiO-66-defect, UiO-66-Cu^{I} and UiO-66-Cu^{II}.

Table S3. Summary of NH_3 adsorption in UiO-66-defect, UiO-66-Cu^{I} and UiO-66-Cu^{II}

MOF	UiO-66-defect	UiO-66-Cu^{I}	UiO-66-Cu^{II}
Pore volume of MOF (cm^3/g)[a]	0.388	0.388	0.388
Uptake capacity of NH_3 (mmol/g)[b]	11.8	12.6	16.9
Storage density of NH_3 in the MOF (g/cm^3)[b]	0.240	0.251	0.337
Packing density of NH_3 in the pore (g/cm^3)[b]	0.517	0.553	0.742
Dynamic uptake of NH_3 (mmol/g)[c]	2.07	3.07	4.15
Enthalpy of adsorption (kJ/mol)	15 to 40	3 to 35	25 to 55
Entropy of adsorption [J/(mol·K)]	-120 to -230	-20 to -180	-130 to -250

[a]: From crystal structure
[b]: At 273K 1 bar
[c]: From breakthrough curves for NH_3 (630 ppm of NH_3 diluted in He, 100 mL min^{-1})

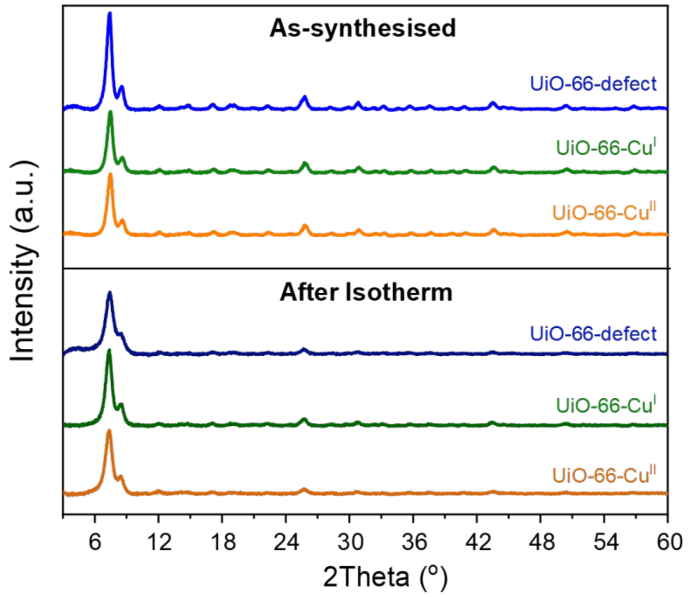

Figure S6. PXRD patterns of as-synthesised materials before and after NH_3 adsorption.

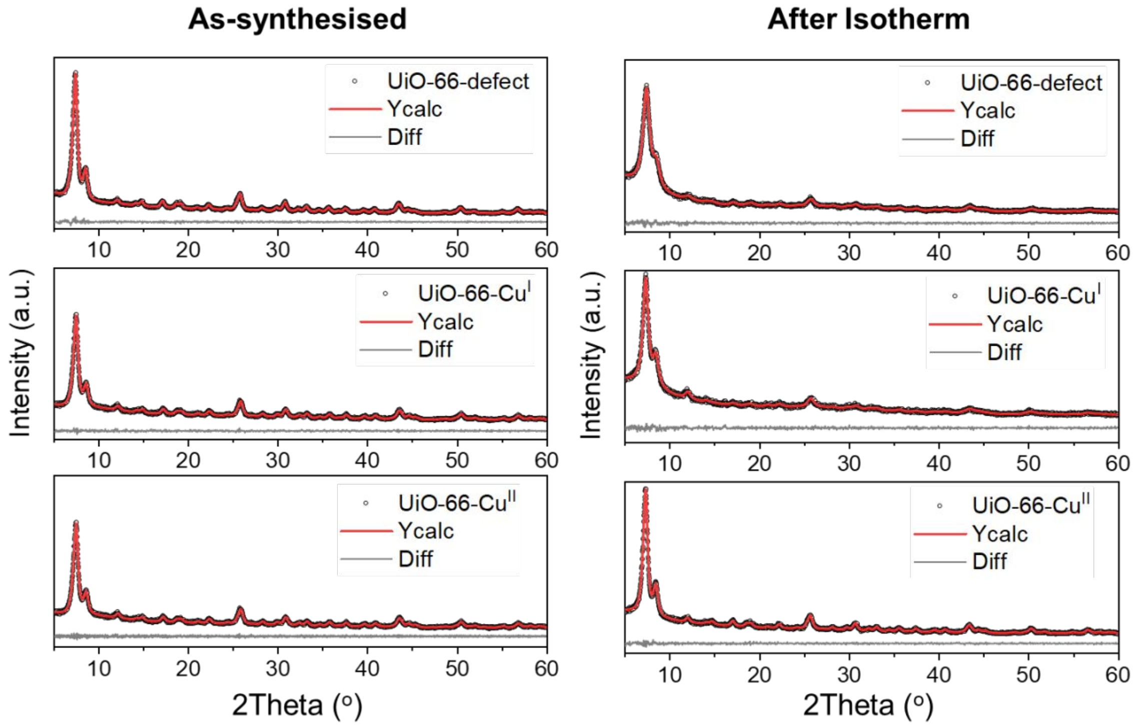

Figure S7. PXRD profiles of Pawley refinements on as-synthesised and after-isotherm UiO-66-defect, UiO-66-Cu^{I} and UiO-66-Cu^{II}.

Table S4. Agreement factors of Pawley refinement on PXRD patterns for UiO-66-defect, UiO-66-Cu^{I} and UiO-66-Cu^{II} before and after NH_3 adsorption.

Materials	As-synthesised			After Isotherm		
	UiO-66-Defect	UiO-66-Cu^{I}	UiO-66-Cu^{II}	UiO-66-Defect	UiO-66-Cu^{I}	UiO-66-Cu^{II}
Crystal System	Cubic	Cubic	Cubic	Cubic	Cubic	Cubic
Space group	*Fm*-3*m*	*Fm*-3*m*	*Fm*-3*m*	*Fm*-3*m*	*Fm*-3*m*	*Fm*-3*m*
a [Å]	20.775(5)	20.760(6)	20.760(6)	20.786(8)	20.781(8)	20.748(8)
Radiation type	In-house PXRD, Cu target, Main wavelength 1.54056 Å					
FWHF of (111) peak	0.693	0.693	0.695	0.935	0.850	0.695
R_{wp}(%)	4.6892	4.5928	4.5933	4.5381	4.6152	4.5930
R_{p}(%)	3.5034	3.4889	3.4899	3.3502	3.4817	3.4666
R_{exp}(%)	4.4007	4.4170	4.4160	4.2001	4.4222	4.3002
Gof	1.065	1.040	1.042	1.080	1.044	1.068

3. Neutron Powder Diffraction (NPD) Patterns

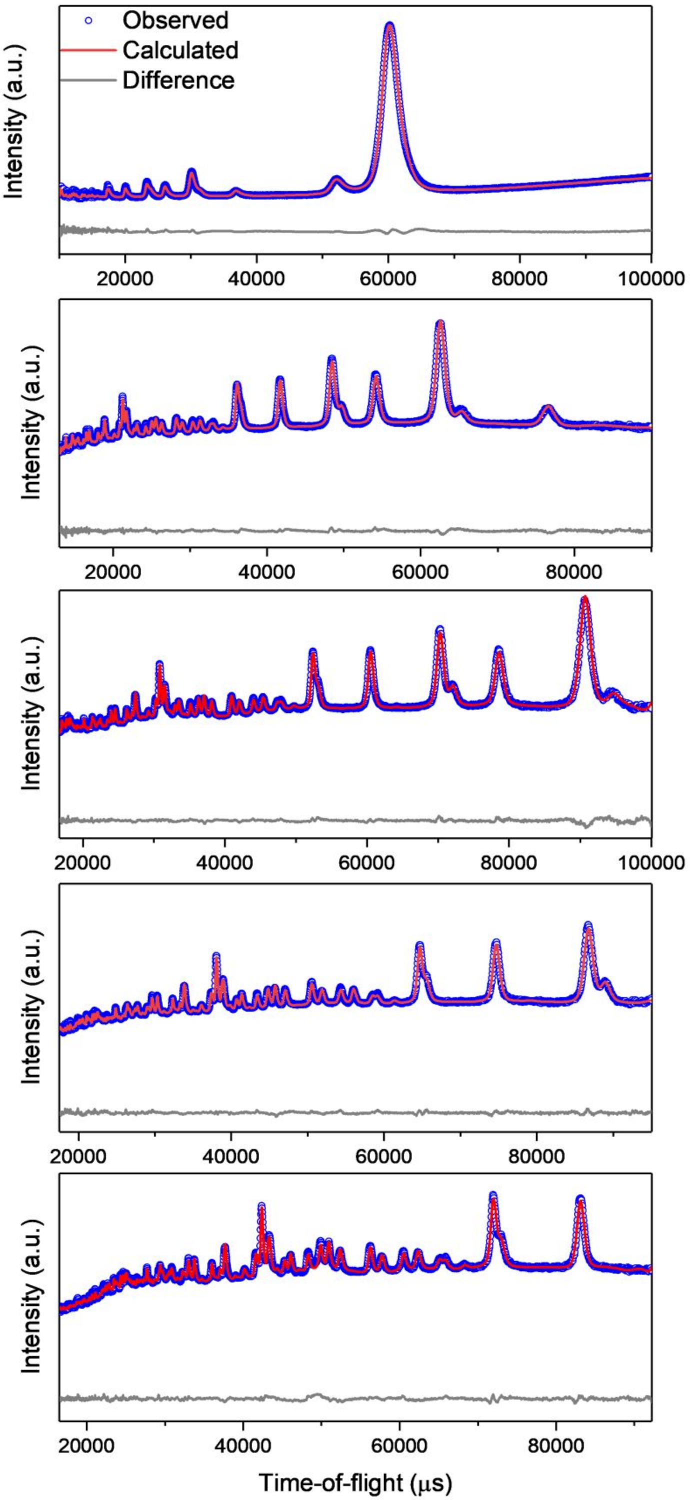

Figure S8. Neutron powder diffraction patterns and Rietveld refinement for bare UiO-66-defect (banks 1 to 5). Fitting agreement parameters: R_{exp}=0.36%; R_{wp}=0.96%; R_p=0.95%; Gof=2.66.

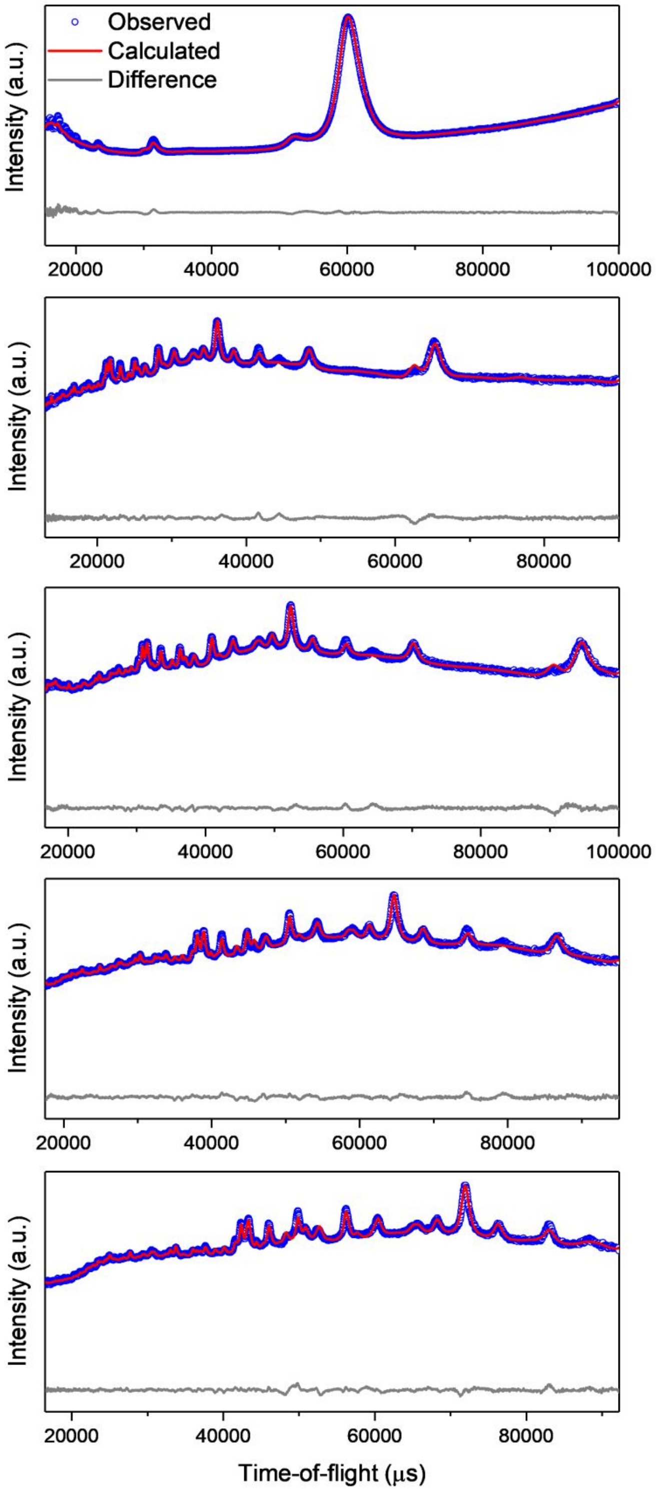

Figure S9. Neutron powder diffraction patterns and Rietveld refinement for UiO-66-defect·10.6ND_3 (banks 1 to 5). Fitting agreement parameters: R_{exp}=0.34%; R_{wp}=0.84%; R_p=0.70%; Gof=2.45.

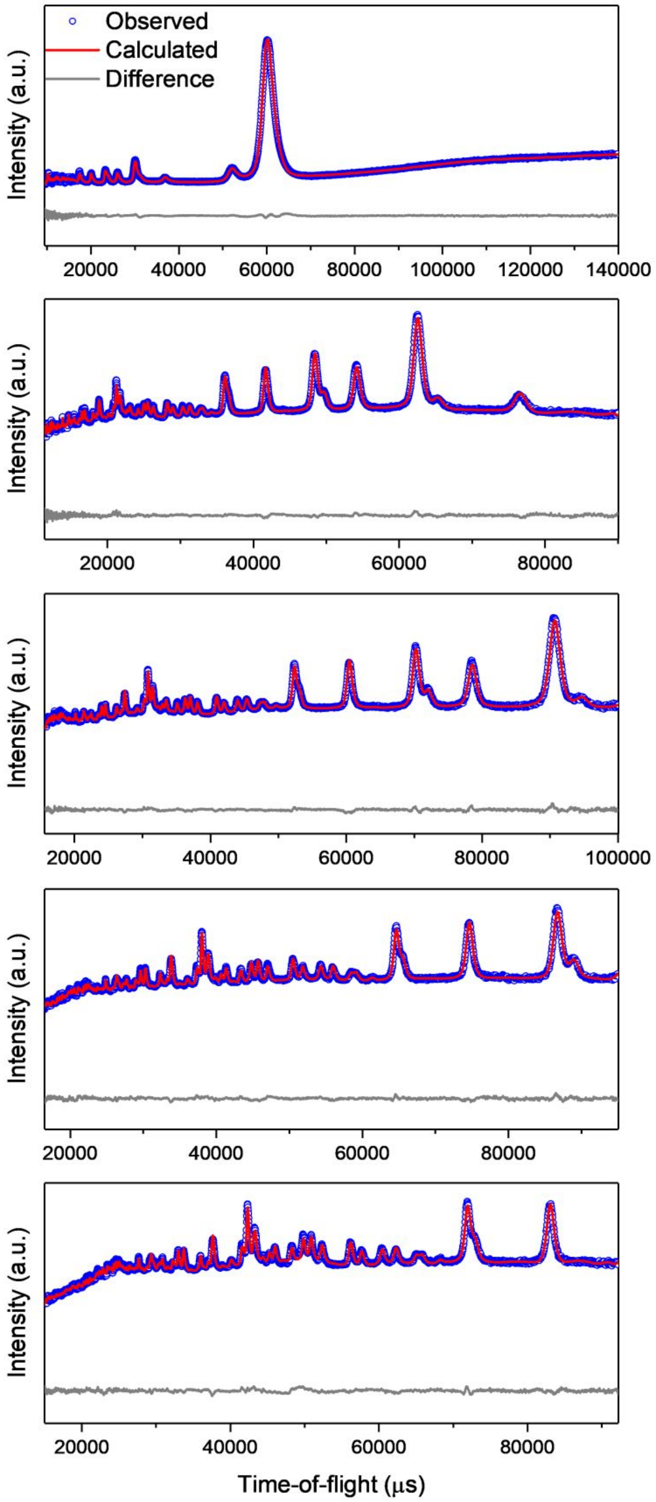

Figure S10. Neutron powder diffraction patterns and Rietveld refinement for bare UiO-66-Cu^{II} (banks 1 to 5). Fitting agreement parameters: R_{exp}=0.45%; R_{wp}=0.94%; R_p=0.90%; Gof=2.09.

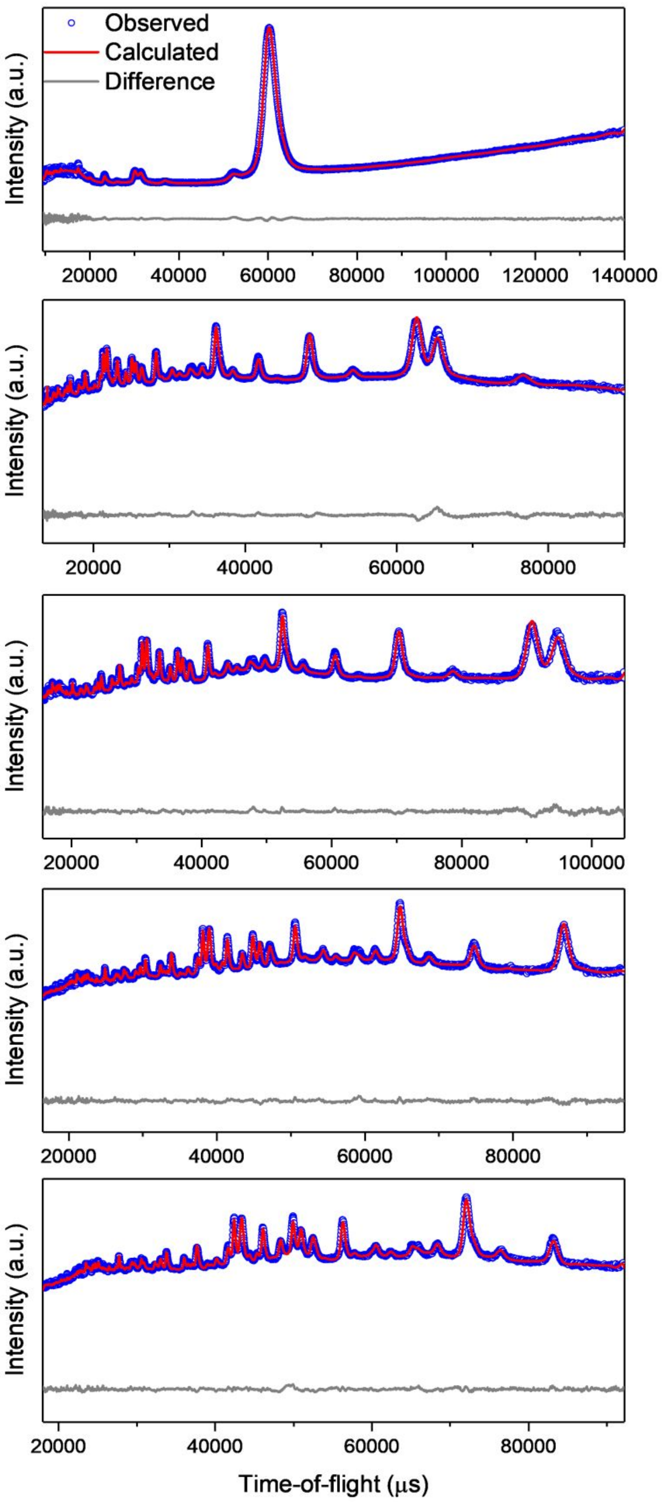

Figure S11. Neutron powder diffraction patterns and Rietveld refinement for UiO-66-$Cu^{II}\cdot 3.34ND_3$ (banks 1 to 5). Fitting agreement parameters: R_{exp}=0.42%; R_{wp}=0.84%; R_p=0.86%; Gof=1.99.

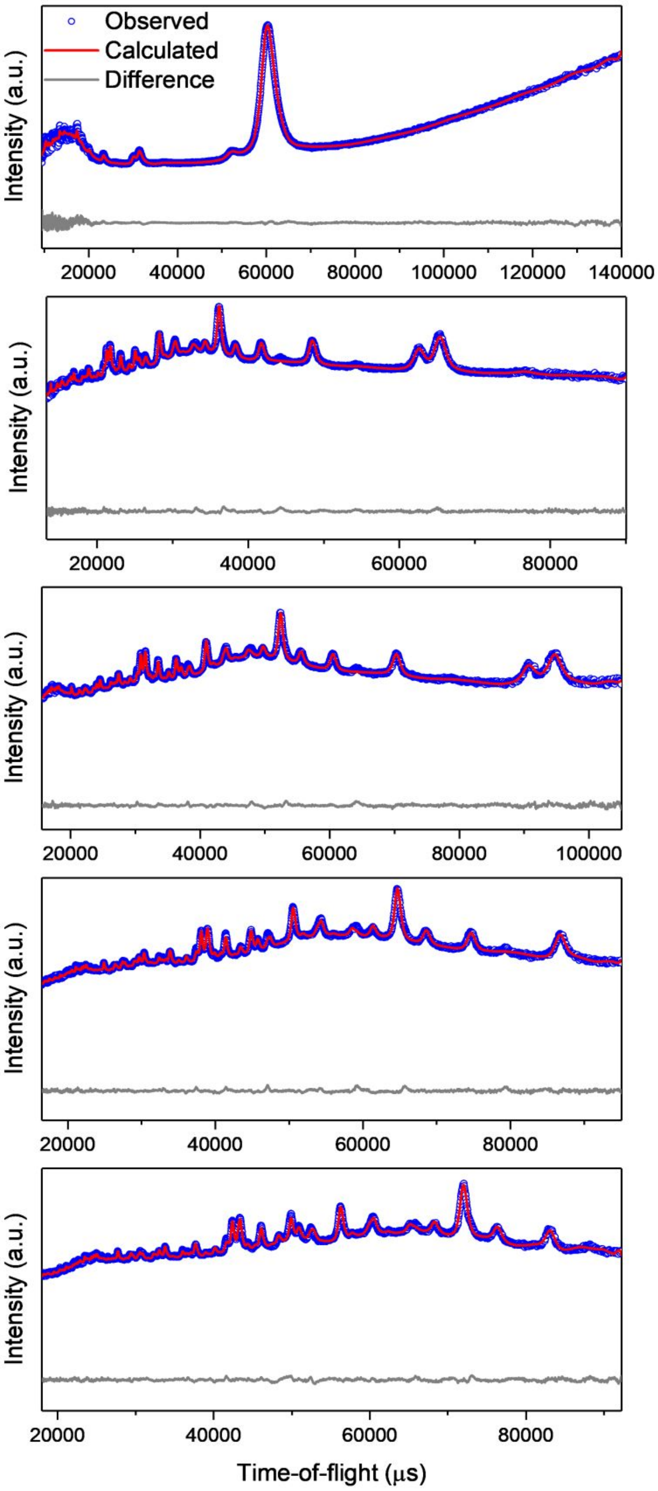

Figure S12. Neutron powder diffraction patterns and Rietveld refinement for UiO-66-$Cu^{II}\cdot 9.64ND_3$ (banks 1 to 5). Fitting agreement parameters: R_{exp}=0.41%; R_{wp}=0.75%; R_p=0.79%; Gof=1.83.

4. Additional Views of Crystal Structures

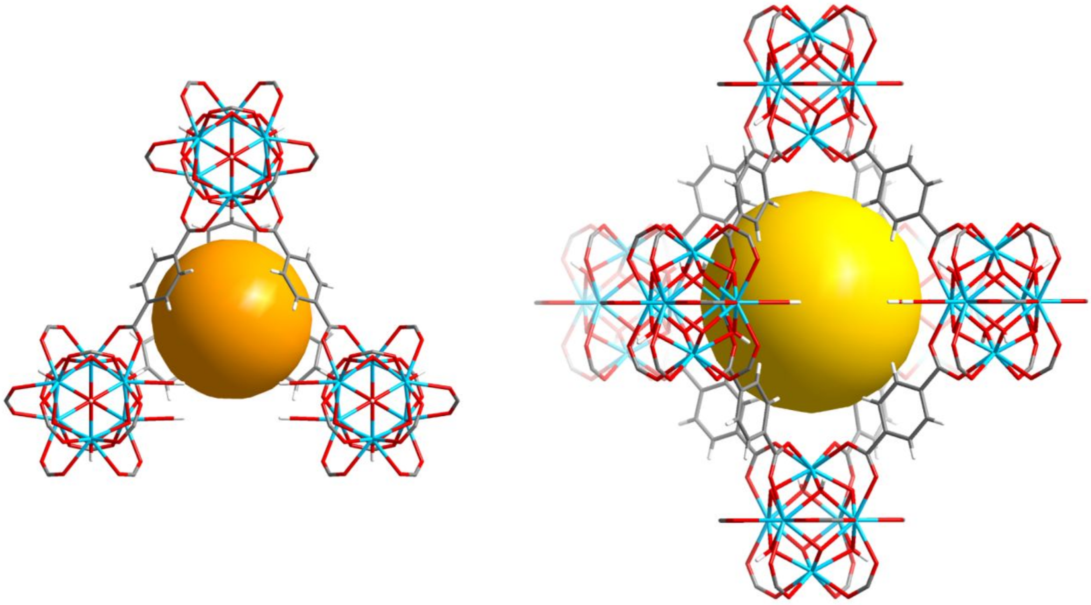

Figure S13. Views of the two types of cages in UiO-66-defect: tetrahedral (left) and octahedral (right) with diameters of 7 Å and 9 Å, respectively.

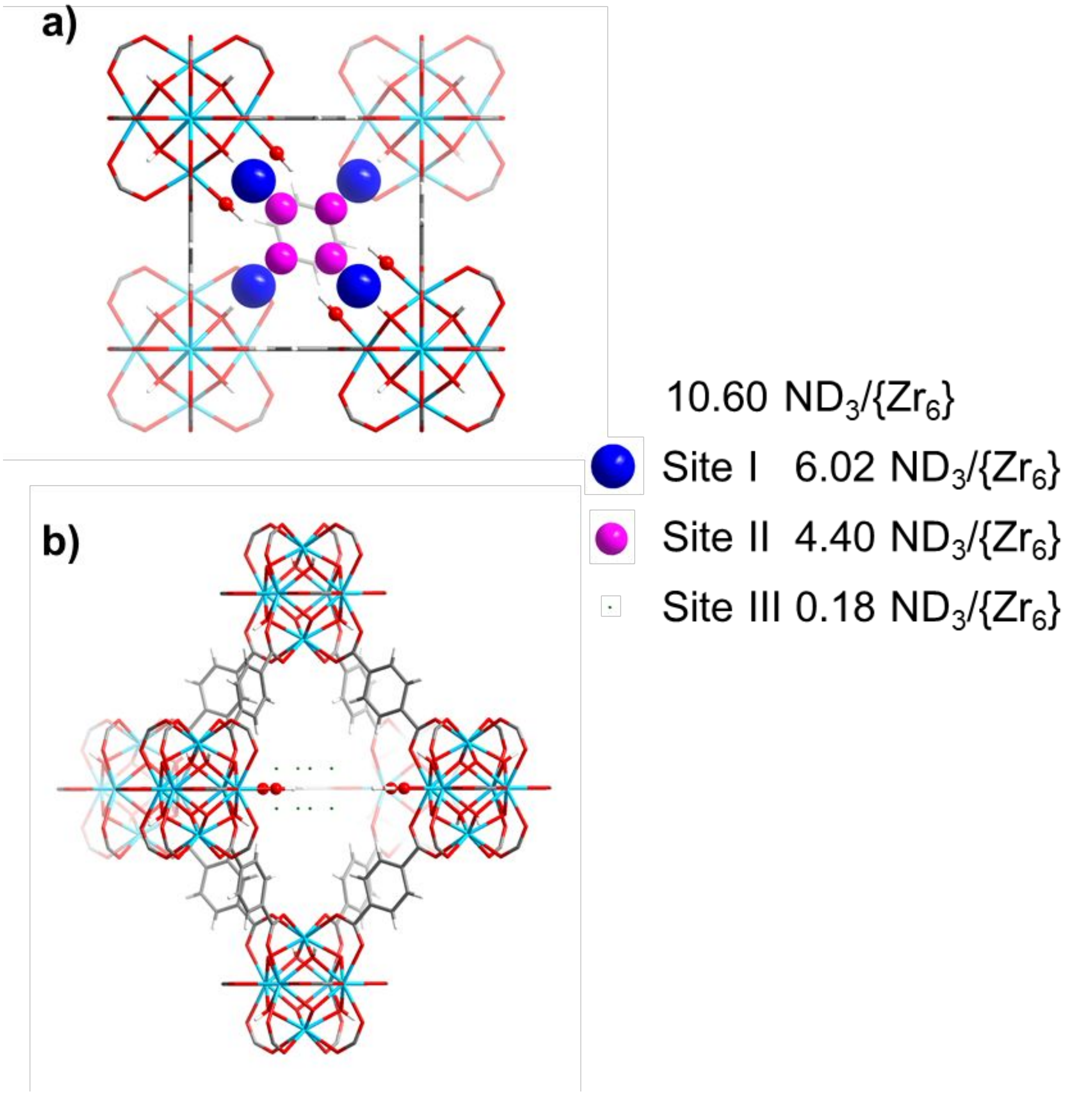

Figure S14. Views of the binding sites for ND_3 in UiO-66-defect·10.6ND_3. Sites in (a) tetrahedral and (b) octahedral cages. (C, grey; O, red; Zr, sky blue; H, white)

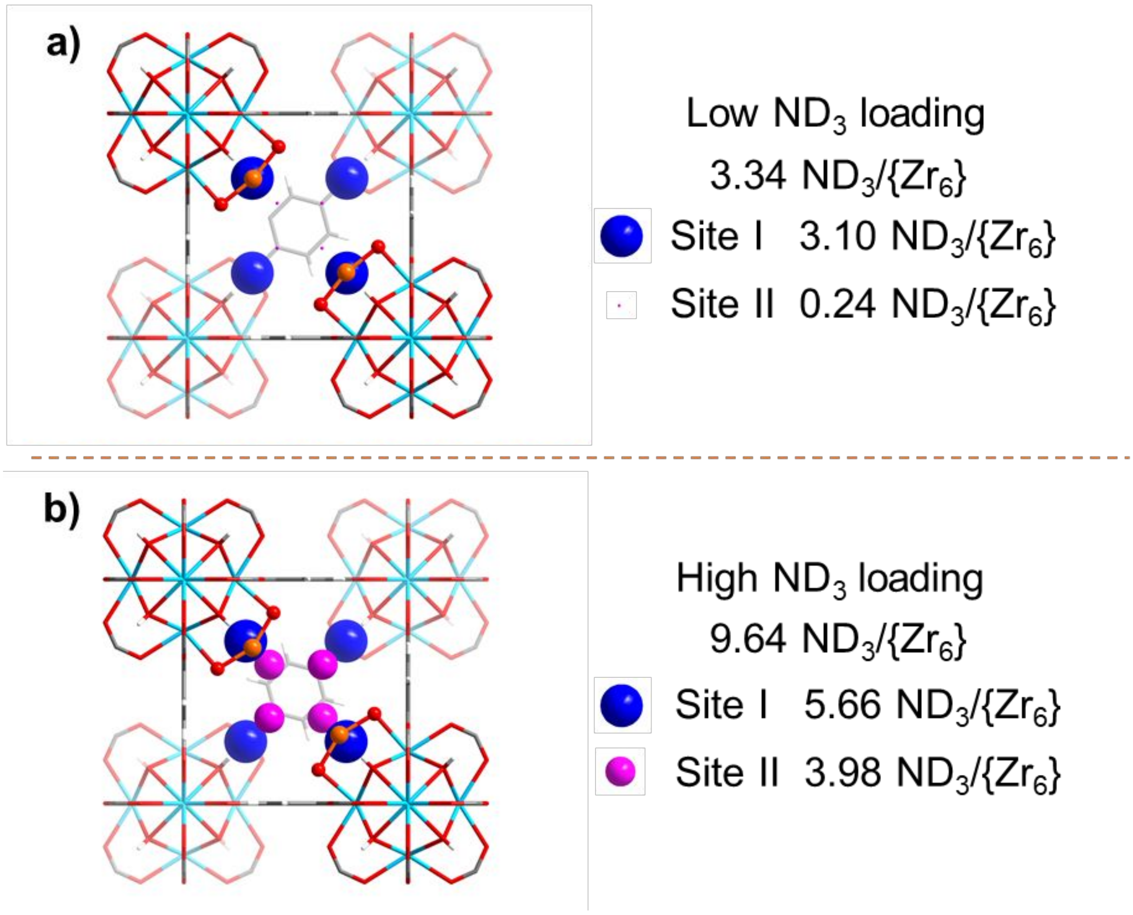

Figure S15. Views of the binding sites for ND_3 in (a) UiO-66-Cu^{II}·3.34ND_3 and (b) UiO-66-Cu^{II}·9.64ND_3 (C, grey; O, red; Zr, sky blue; H, white; Cu, orange).

5. Infrared and UV-vis Spectroscopy

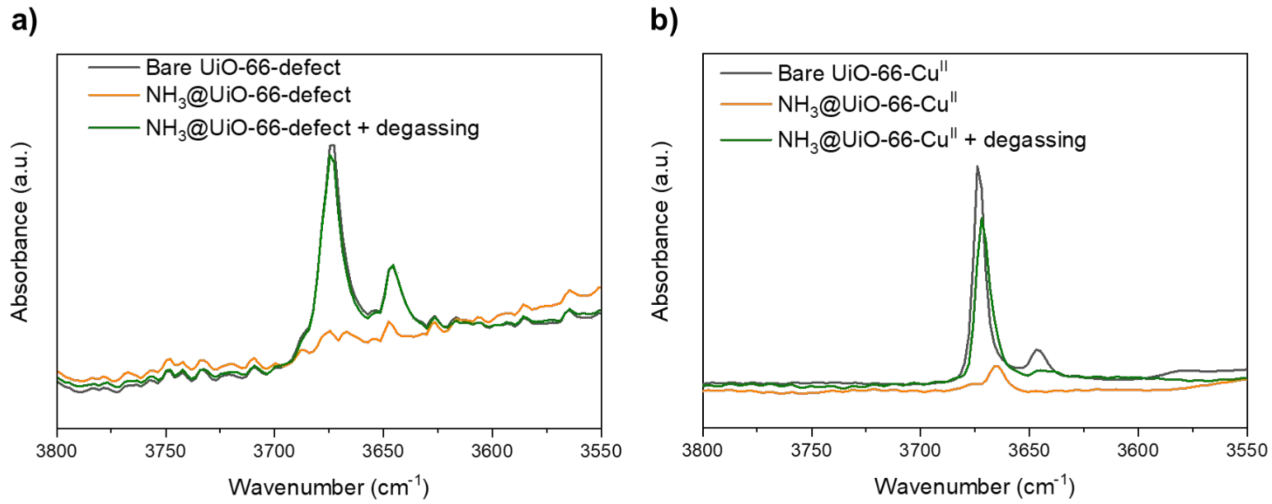

Figure S16. *In situ* infrared spectra of (a) UiO-66-defect and (b) UiO-66-Cu^{II} upon adsorption and desorption of NH_3. The infrared spectra were recorded at a resolution of 4 cm^{-1}. The spectrum of background KBr has been subtracted.

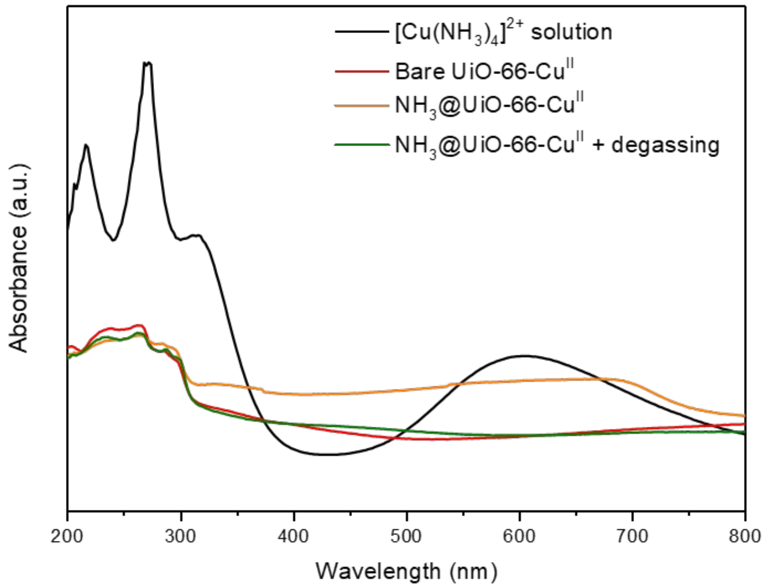

Figure S17. UV-vis spectra of $[Cu(NH_3)_4]^{2+}$ solution, bare UiO-66-Cu^{II}, NH_3@UiO-66-Cu^{II}, and NH_3@UiO-66-Cu^{II} after degassing.

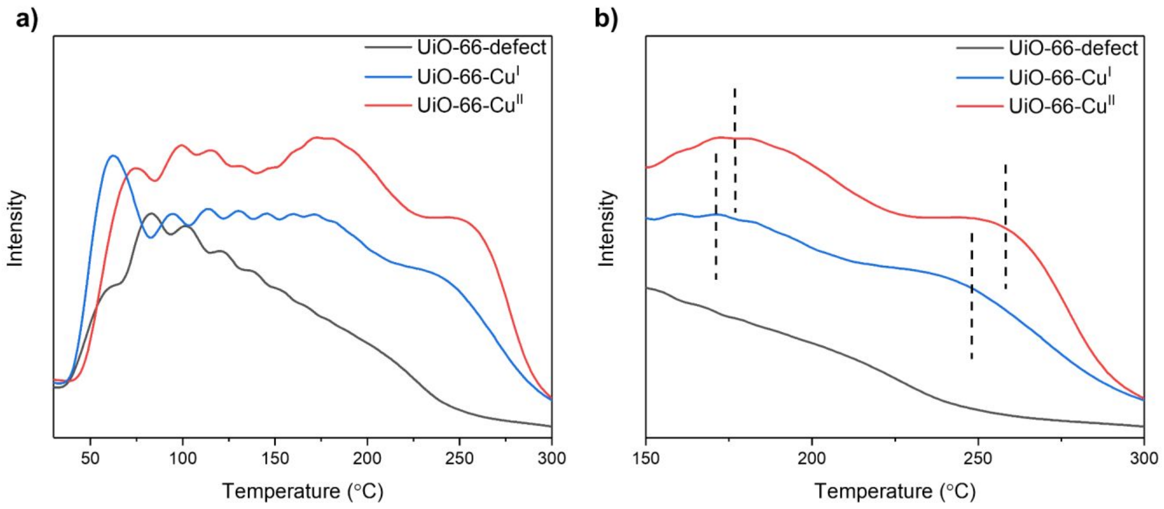

Figure S18. NH_3-TPD plots of UiO-66-defect, UiO-66-Cu^I, and UiO-66-Cu^{II}. Section of the plots in (a) between 150−300 °C is shown in (b).

6. Solid-State Nuclear Magnetic Resonance (NMR) Spectroscopy

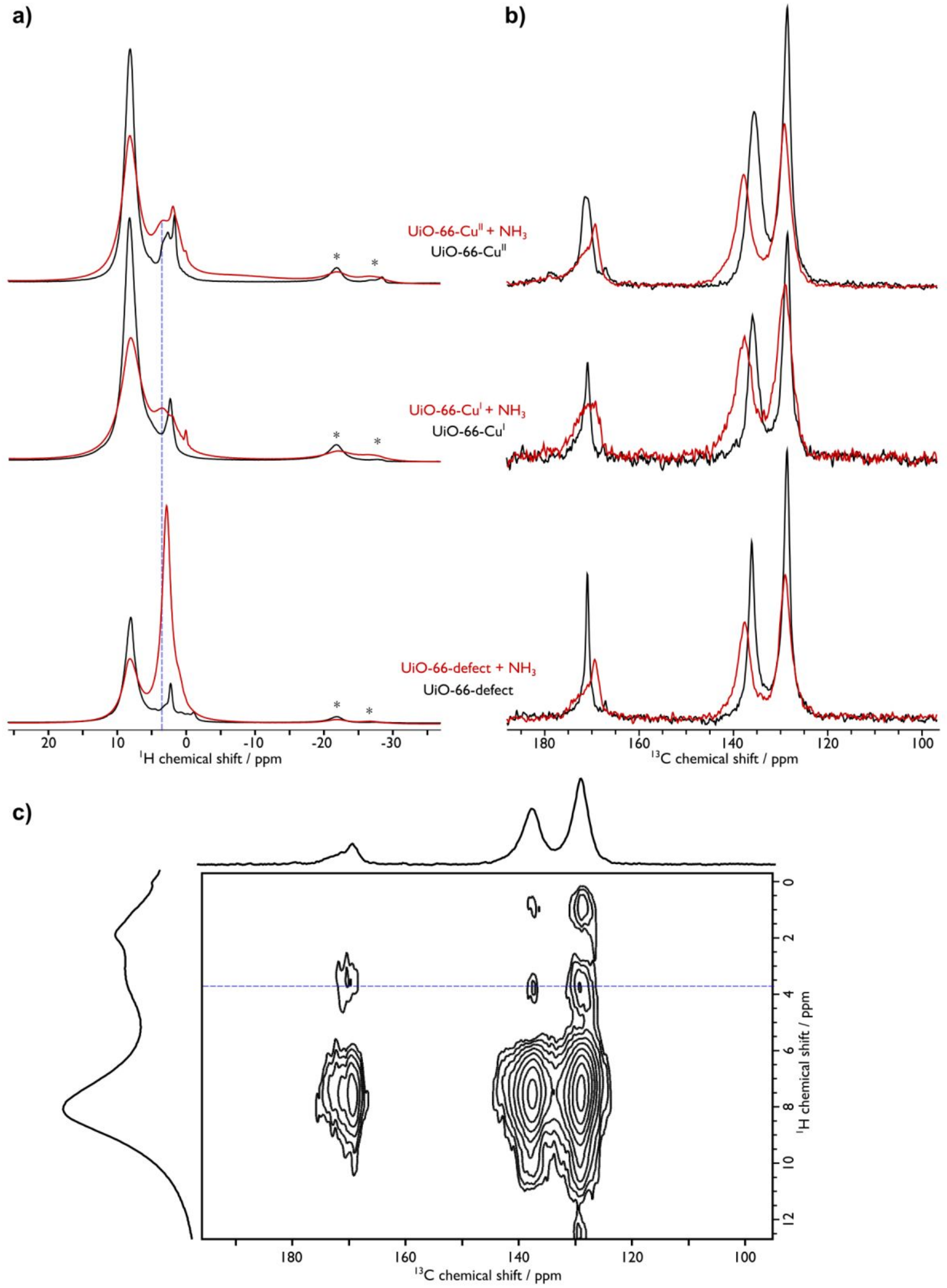

Figure S19. (a) ^{1}H Hahn-echo and (b) {^{1}H-}^{13}C CP MAS NMR spectra of bare (black) and NH_3-loaded (red) UiO-66-defect (bottom), UiO-66-Cu^I (middle), and UiO-66-Cu^{II} (top). (c) ^{1}H-^{13}C FSLG-HETCOR MAS NMR spectrum of NH_3-loaded UiO-66-Cu^{II} and corresponding ^{1}H DEPTH (left) and {^{1}H-}^{13}C CP (top) MAS NMR spectra. All spectra were recorded at 9.4 T using a MAS frequency of 12 kHz. The dashed blue lines highlight the signal from confined NH_3 in the UiO-66-Cu^I and UiO-66-Cu^{II}. Asterisks denote the position of spinning side bands.

7. Electron Paramagnetic Resonance (EPR) Spectroscopy

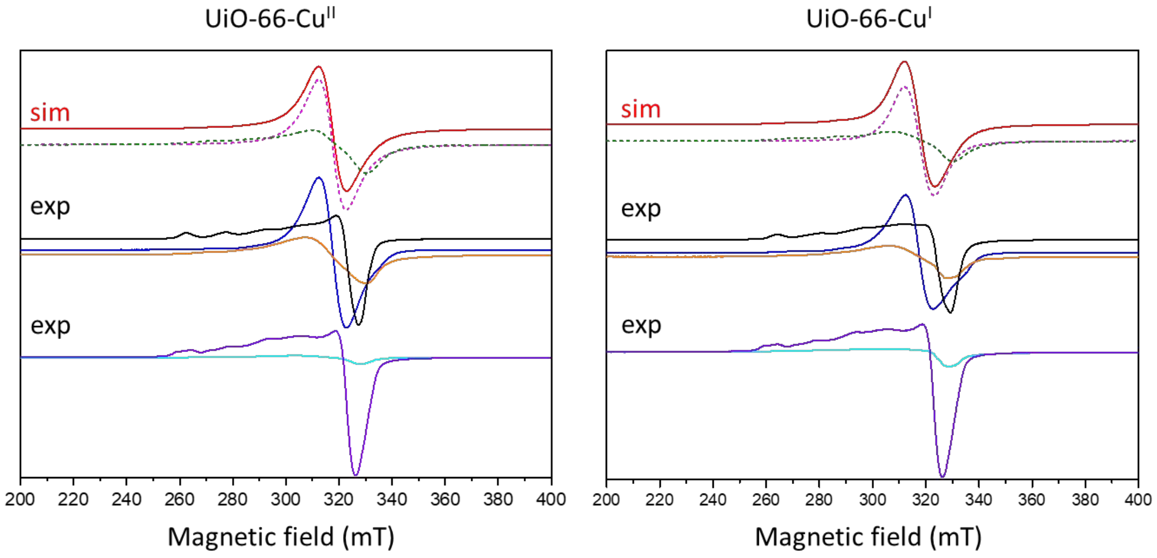

Figure S20. Experimental and simulated X-band (9.4 GHz) EPR spectra of UiO-66-Cu^{II} and UiO-66-Cu^{I} at 40 K. Blue: experimental spectra of samples after NH_3 adsorption; orange: experimental spectra of samples after desorption of NH_3; black: experimental spectra of samples after desorption of NH_3 and exposure to the air for 24 h; purple: experimental spectra of pre-activated solvated form of samples; cyan: experimental spectra of activated samples before adsorption of NH_3; magenta: simulated spectra of unresolved broad signal; green: simulated spectra of axial monomeric Cu(II); red: simulated spectra with sum of both Cu components. The simulated spectra shown here are corresponding to the spectra of both samples after adsorption of NH_3. Parameters of simulation are shown in Table S5.

Table S5. EPR simulation parameters of EPR spectra of NH_3@UiO-66-Cu^{II} and NH_3@UiO-66-Cu^{I} (blue spectra in Figure 5b and S20).

NH_3@UiO-66-Cu^{II}				
Species	weight	g-tensor	$^{63/65Cu}$A tensor/MHz	Linewidth/mT
broad signal	0.95	2.115	-	[4 16]
isolated Cu	0.05	[2.07, 2.11, 2.27]	[-, -, 530]	[0 14]
NH_3@UiO-66-Cu^{I}				
Species	weight	g-tensor	$^{63/65Cu}$A tensor/MHz	Linewidth/mT
broad signal	0.9	2.115	-	[4 16]
isolated Cu	0.1	[2.07, 2.11, 2.27]	[-, -, 530]	[0 14]

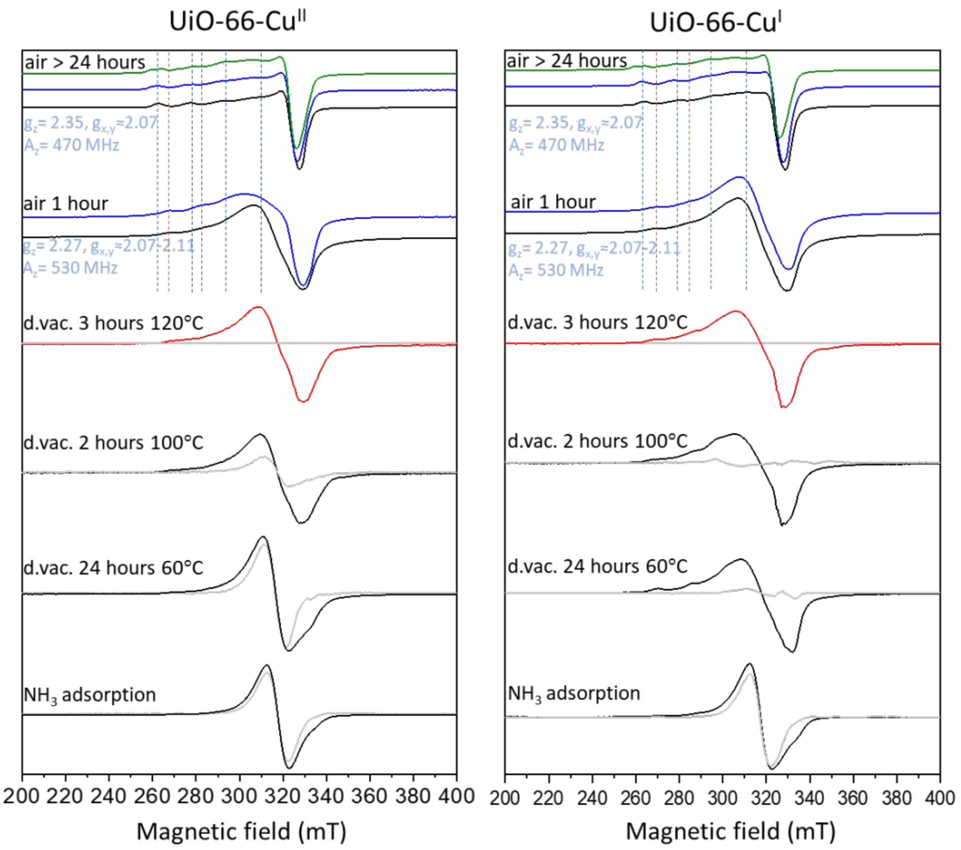

Figure S21. X-band (9.4 GHz) EPR spectra (scaled to maximum of intensity) of UiO-66-Cu^{II} and UiO-66-Cu^{I} at 40 K: as-synthesised sample (green); sample activated, reduced and exposed to the air (blue); sample after desorption of NH_3 and exposure to air (black, red, and grey): black, spectra under annotated conditions; red, experimental spectra after final degassing for 3 h under 120 °C heating, where only the anisotropic isolated Cu signal remains; grey, spectra resulting from subtraction of the isolated Cu signal *i.e.*, residual of unresolved broad signal.

The EPR spectra recorded during the NH_3 degassing process described in the main text is shown in Figure S21. The spectra show how Cu sites recover to the original axial monomeric Cu(II) centre on re-activation and exposure to air. UiO-66-Cu^{I} and UiO-66-Cu^{II} after desorption of NH_3 and exposure to air give identical spectra (simulated with same spin Hamiltonian parameters) compared with the initial UiO-66-Cu^{II} material, further proving the high stability and reusability of the materials.

Figure S21 illustrates the relative quantity of the unresolved broad signal and isolated Cu(II) signal during the NH_3 degassing process. The black spectra are the experimental data recorded at different re-activation (degassing) conditions. Spectra recorded after final degassing process (dynamic vacuum and 3 h of heating to 120 °C) is shown in red and refers to isolated Cu signal. The grey spectra were calculated from: spectrum (grey) = spectrum (black, partially NH_3-desorbed sample) – (weight %) * spectrum (red, isolated Cu(II)), giving the residual of the unresolved broad signal. Thus, the relative amount of two different Cu components can be estimated by: (i) for unresolved broad signal (square shaped in Figure 5d): [second integral of spectrum (grey) / second integral of spectrum (black)] under the same conditions; (ii) for isolated Cu(II) (circle shaped

in Figure 5d): 1 – [second integral of spectrum (grey) / second integral of spectrum (black)] under the same conditions. The resultant values for different component contribution are shown on Figure 5d.

By EPR we observe a difference in NH_3 desorption between UiO-66-Cu^{II} and UiO-66-Cu^{I}. The remaining isolated Cu(II) signal is retained in both samples until prolonged exposure to air where it is converted to the original hydrated Cu(II) form (also shown by HYSCORE, Figure S23). This EPR analysis is consistent with the selective desorption of the NH_3 molecules at Site II/III.

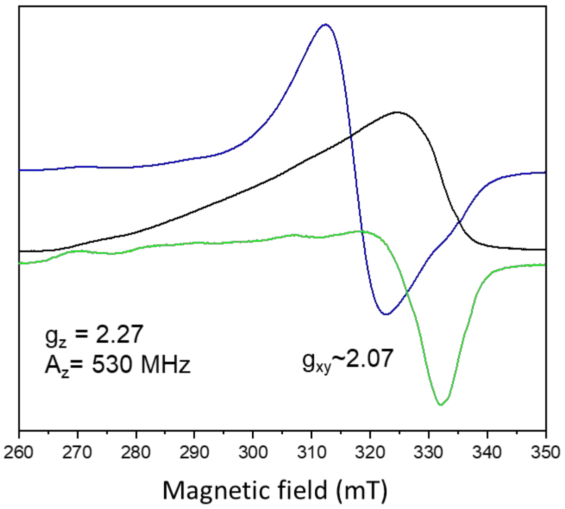

Figure S22. X-band (9.4 GHz) EPR spectra of UiO-66-Cu^{I} at 6 K after NH_3 loading; blue: CW spectrum; black: echo-detected spectra recorded with π/2=16 ns and tau=150 ns; green: derivative of echo-detected spectra recorded with π/2=16 ns and tau=150 ns.

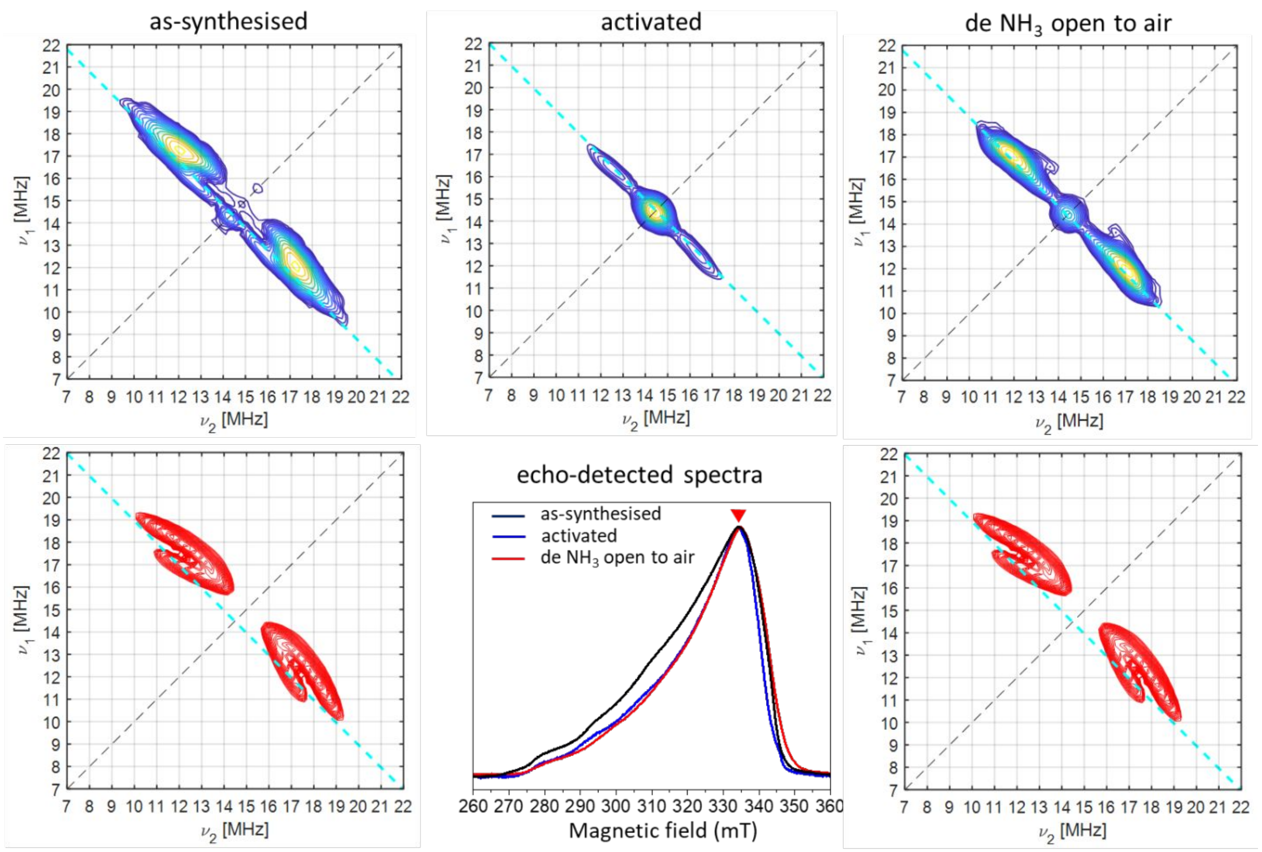

Figure S23. Upper row (left to right): X-band (9.7368) ^{1}H HYSCORE spectra of as-synthesised UiO-66-Cu^{II} (left), activated UiO-66-Cu^{II} (middle) and UiO-66-Cu^{II} (right) after desorption of NH_3 and exposure to air, measured at the same static magnetic field of 340 mT (recorded at 5 K). Bottom row (left and right): simulated spectra (Table S6); middle: echo-detected (ED) EPR spectra of UiO-66-Cu^{II} samples.

The local environment of the Cu(II) in the as-synthesised/NH_3-degassed UiO-66-Cu^{II} was investigated by hyperfine sublevel correlation (HYSCORE) spectroscopy.[11] The simulated spectra were modelled by considering contributions from the electron-nuclear (^{1}H) dipolar and isotropic hyperfine interactions: $A = A_{\text{dip}} + A_{\text{iso}}$. The dipolar ^{1}H hyperfine interaction matrix is defined with the local z-axis directed along the Cu(II)−H vector and calculated in the high-field approximation as $A_{\text{dip}} = [-T;-T;+2T]$. Here, $T = \mu_0 g_e g_n \mu_e \mu_n / 4\pi r^3$, where μ_0 is the vacuum permeability, μ_n is the nuclear magneton, g_n is the nuclear g-factor and r is the Cu···H distance. Transformation of the $\boldsymbol{A}$ matrix into the molecular frame, defined by the g-tensor frame of the Cu(II) site (X||g_{xx}, Y||g_{yy}, Z||g_{zz}), used a single Euler angle corresponding to a rotation about the Ay axis, *i.e.*, rotating the Cu−H vector into the g_{xz} plane.

The surrounding ^{1}H nuclei of as-synthesised UiO-66-Cu^{II} was investigated in a previous paper.[1] The ^{1}H HYSCORE gives a pair of ridges shifted away from the anti-diagonal in the (+,+) quadrant, which was assigned to protons from H_2O coordinated in the equatorial (i.e. g_{xy}) plane of a Cu(II) ion. The spectrum of UiO-66-Cu^{II} after NH_3 desorption and exposure to air shows the same proton binding sites as the as-synthesised sample in the ^{1}H HYSCORE spectra. A good simulation is achieved with the hyperfine parameters $A_{\text{iso}} = -1$ MHz, $T =$

+6.2 MHz where we fixed the Euler angles [α, β, γ] = [0, 90, 0]. These results indicate that the UiO-66-Cu^{II} is fully restored with binding of structural H_2O to the Cu site.

Table S6. Simulation parameters of Pulse ^{1}H HYSCORE EPR spectra at X-band of UiO-66-Cu^{II} (as-synthesised and fully degassed) shown in Figure S23.

Nuclei	Electron Spin	Nuclei Spin	g-tensor	Euler angle (degree)	Aiso (MHz)	T (MHz)	Distance (Å)
^{1}H	1/2	1/2	[2.07 2.07 2.35]	[0 90 0]	-1	6.2	2.3

Table S7. Summary of the isothermal NH_3 adsorption capacities and details of selected MOF materials.

Name	Uptake (mmol g^{-1})	Condition	Stability towards Dry Ammonia	Reference
[Mg_2(dobpdc)]	23.9	298K 1bar	Reversible for 3 Cycles	14
[Ni_2(dobpdc)]	20.8	298K 1bar	Reversible for 3 Cycles	14
[Cu_2Cl_2(BBTA)]	19.8	298K 1bar	Loss of Crystallinity	15
[Co_2Cl_2BBTA]	17.9	298K 1bar	Reversible for 3 Cycles	15
Fe-MIL-101-SO_3H	17.8	298K 1bar	Not Discussed	16
[$Zn_2(L_1)_2$(bpe)]	17.8	293K 1bar	Not Discussed	17
[Co$(NA)_2$]	17.5	298K 1bar	Loss of Crystallinity	18
MFM-300(V^{IV})	17.3	273K 1bar	Reversible for 18 cycles	19
UiO-66-Cu^{II}	16.9	273K 1bar	Reversible for 15 cycles	This Work
DUT-6-$(OH)_2$	16.4	298K 1bar	Loss of Crystallinity	20
Mg-MOF-74	16.2	298K 1bar	Stable up to 350 °C	21
MFM-300(V^{III})	16.1	273K 1bar	Reversible for 24 cycles	19
MFM-300(Al)	15.7	273K 1bar	Reversible for 50 cycles	22
MFM-300(Fe)	15.6	273K 1bar	Reversible for 20 cycles	19
[Mn_2Cl_2BTDD]	15.5	298K 1bar	Reversible for 3 Cycles	23
[Zn_2(dobpdc)]	15.2	298K 1bar	Loss of Crystallinity	14
Fe-soc-MOF	14.7	298K 1bar	Decrease of Uptake	24
[Ni_2Cl_2BBTA]	14.7	298K 1bar	Reversible for 3 Cycles	15
[$Zn_2(L^1)_2$(bipy)]	14.3	293K 1bar	Not Discussed	17
MFM-300(Cr)	14.0	273K 1bar	Reversible for 35 cycles	19
[Cu$(NA)_2$]	13.4	298K 1bar	Loss of Crystallinity	18
[Co_2(dobpdc)]	13.3	298K 1bar	Reversible for 3 Cycles	14

[Mn_2(dobpdc)]	13.3	298K 1bar	Reversible for 3 Cycles	14
MFM-300(Sc)	13.1	298K 1bar	Reversible for 5 Cycles	38
UiO-66-Cu^{I}	12.6	273K 1bar	Reversible for 15 cycles	This Work
MOF-5	12.2	298K 1bar	Loss of Crystallinity	25
MOF-177	12.2	298K 1bar	Loss of Crystallinity	25
HKUST-1	12.1	301K 1.2 bar	Loss of Crystallinity	26
[Ni_2(adc)$_2$(dabco)]	12.1	295K 1bar	Reversible for 3 Cycles	27
[Ni_2Cl_2BTDD]	12.0	298K 1bar	Reversible for 3 Cycles	23
[Co_2Cl_2BTDD]	12.0	298K 1bar	Loss of Crystallinity	23
DUT-6	12.0	298K 1bar	Loss of Crystallinity	20
UiO-66-defect	11.8	273K 1bar	Reversible for 15 cycles	This Work
[Co_2(adc)$_2$(dabco)]	11.2	295K 1bar	Reversible for 3 Cycles	27
Ga-PMOF	10.5	298K 1bar	Loss of Crystallinity	28
Zn(NA)$_2$	10.2	298K 1bar	Structural Change	18
MIL-101	10.0	298K 1bar	Reversible for 5 Cycles	29
MFM-303	9.9	273K 1bar	Reversible for 30 Cycles	39
In-PMOF	9.4	298K 1bar	Decrease of Uptake	28
UiO-bpydc	8.4	298K 1.1bar	Stable for 1 Cycle	30
NU-1401	8.4	298K 1bar	Stable for 1 Cycle	31
UiO-67	8.4	298K 1.1bar	Stable for 1 Cycle	30
[Zn_2(adc)$_2$(dabco)]	8.3	295K 1bar	Reversible for 3 Cycles	27
NU-300	8.3	298K 1bar	Loss of Crystallinity	32
UiO-66-C	8.3	298K 1bar	Stable for 1 Cycle	33
MIL-100	8.0	298K 1bar	Reversible for 5 Cycles	29

NU-1000-Cl-120	7.8	298K 1bar	Reversible for 3 Cycles	34
NU-1000-F-120	7.8	298K 1bar	Reversible for 3 Cycles	35
Al-PMOF	7.7	298K 1bar	Reversible for 2 Cycles	28
NU-1000-F-60	7.6	298K 1bar	Reversible for 3 Cycles	35
NU-1000-Cl-300	7.1	298K 1bar	Reversible for 3 Cycles	34
NDC-NU-1000-F-120	6.9	298K 1bar	Reversible for 3 Cycles	35
UiO-66-B	6.8	298K 1bar	Stable for 1 Cycle	33
NDC-NU-1000-F-60	6.8	298K 1bar	Reversible for 3 Cycles	35
ECUT-36	6.6	273K 1bar	Reversible for 2 Cycles	36
$[Cu_2(adc)_2(dabco)]$	6.5	295K 1bar	Loss of Crystallinity	27
NDC-NU-1000-FF-60	6.2	298K 1bar	Reversible for 3 Cycles	35
$[Cd(NA)_2]$	6.0	298K 1bar	Structural Change	18
Al-BTB	6.0	298K 1bar	Stable up to 350 °C	21
NU-1000-FF-300	5.9	298K 1bar	Reversible for 3 Cycles	35
NU-1000-FF-60	5.8	298K 1bar	Reversible for 3 Cycles	35
NDC-NU-1000-FF-120	5.8	298K 1bar	Reversible for 3 Cycles	35
UiO-66-A	5.7	298K 1bar	Stable for 1 Cycle	33
SION105-Eu	5.7	303K 1bar	Reversible for 5 Cycles	37
NU-1000-FF-120	5.5	298K 1bar	Reversible for 3 Cycles	35
NU-1000-F-300	5.4	298K 1bar	Reversible for 3 Cycles	35
NH_2-MIL-53	5.4	298K 1bar	Reversible for 5 Cycles	29
MIL-53	4.4	298K 1bar	Reversible for 5 Cycles	29

Table S8. Summary of the dynamic NH_3 adsorption capacities in selected MOF materials below 1 mbar.

Name	Uptake (mmol/g)	Measuring Condition	Reference
Co_2Cl_2BBTA	8.56	298 K 1.0 mbar	15
Mg_2(dobpdc)	8.25	298 K 0.57 mbar	14
Cu_2Cl_2BBTA	7.52	298 K 1.0 mbar	15
IRMOF-3	6.16	298 K 0.99 mbar	40
Ni_2(dobpdc)	5.16	298 K 0.58 mbar	14
MOF-199	5.11	298 K 0.99 mbar	40
Zn_2(dobpdc)	4.98	298 K 0.42 mbar	14
Co_2Cl_2BTDD	4.78	298 K 1.0 mbar	15
Mn_2(dobpdc)	4.77	298 K 0.36 mbar	14
Co_2(dobpdc)	4.72	298 K 0.54 mbar	14
UiO-66-Cu^{II}	4.15	298 K 0.63 mbar	This Work
Fe-MIL-101-SO_3H	3.52	298 K 0.51 mbar	16
UiO-66-Cu^{I}	3.07	298 K 0.63 mbar	This Work
MFM-303	2.9	298K 0.83 mbar	39
UiO-66-NH_3Cl	2.64	298 K 0.66 mbar	16
MOF-177	2.47	298 K 0.99 mbar	40
UiO-66-defect	2.07	298K 0.63mbar	This Work
MFM-300(V^{III})	1.9	298 K 1.0 mbar	19
IRMOF-62	1.35	298 K 0.99 mbar	40
MFM-300(Cr)	1.1	298 K 1.0 mbar	19
MFM-300(V^{IV})	1.0	298 K 1.0 mbar	19
UiO-66-NH_2	0.93	298 K 0.49 mbar	16

MFM-300(Fe)	0.6	298 K 1.0 mbar	19
MOF-5	0.35	298 K 0.99 mbar	40

Table S9. Adsorption data for NH_3 in UiO-66-Cu^{II}

Temperature	273K		283K		293K		303K		313K	
Content	Pressure (mbar)	Uptake (mmol/g)	Pressure (mbar)	Uptake (mmol/g)	Pressure (mbar)	Uptake (mmol/g)	Pressure (mbar)	Uptake (mmol/g)	Pressure (mbar)	Uptake (mmol/g)
Adsorption	0.499075	2.713868	0.495157	1.237408	0.49993	3.005831	0.500577	2.658729	0.50054	2.273643
	0.99509	3.381664	0.994711	1.729003	0.998447	3.237356	0.998654	2.88087	0.998801	2.491492
	1.398593	3.783325	1.399057	1.955571	1.399472	3.365946	1.400022	3.005618	1.398667	2.610108
	1.888933	4.109546	1.911518	2.106136	1.872453	3.469091	1.878556	3.107529	1.866349	2.702932
	2.473686	4.400522	2.457816	2.242875	2.469413	3.585406	2.451712	3.214223	2.499322	2.81376
	4.981773	4.974776	4.981773	2.664884	4.989708	3.895731	4.971397	3.533297	4.990319	3.119035
	9.979027	5.690811	9.980247	3.128962	9.996118	4.266062	9.996728	3.886077	9.973533	3.458571
	14.95248	6.241762	14.99581	3.448798	14.96346	4.520284	14.96835	4.115368	14.96163	3.669964
	20.00222	6.704856	19.99978	3.703339	19.97903	4.721911	19.96621	4.294315	19.97903	3.83225
	49.9894	8.281955	49.95888	4.640721	49.98513	5.494376	49.98147	4.944688	49.94424	4.398699
	74.96041	9.324427	74.98238	5.338717	74.93782	5.990023	74.95675	5.342328	74.97506	4.740062
	97.21683	10.06748	98.54698	5.936864	97.48283	6.344187	96.81776	5.621226	98.81299	4.966097
	117.9672	10.67622	122.6228	6.494369	120.0955	6.68057	116.7701	5.843738	120.4946	5.163757
	141.511	11.27097	147.2307	6.996295	145.3684	7.031955	137.5205	6.089395	140.1808	5.373276
	163.9906	11.80694	172.7697	7.458732	169.3112	7.335796	158.67	6.322877	168.9122	5.569538
	186.8693	12.3028	195.5153	7.84283	192.0569	7.597234	178.8884	6.538644	183.6769	5.740532
	231.4295	13.11602	246.1943	8.52027	241.5387	8.047698	222.5175	6.908127	242.4698	6.049354
	319.4858	14.16242	316.9586	9.353142	309.9087	8.600352	320.0179	7.592625	307.7805	6.443464
	396.2358	14.6356	389.8511	10.15218	380.806	9.095426	397.0338	8.0499	393.0434	6.926988
	495.9975	15.03641	488.9477	11.0676	472.3207	9.625849	499.855	8.517704	479.6366	7.276694

	574.4766	15.38008	584.3198	11.74354	584.0538	10.06049	591.2366	8.927733	589.1083	7.723758
	657.4784	15.69056	678.2288	12.10963	695.6538	10.40667	688.8701	9.243324	686.7418	8.073353
	795.4155	16.07968	772.8028	12.34887	792.3561	10.70546	797.4107	9.581069	794.6174	8.394404
	890.1226	16.54519	899.4337	12.598	896.7733	10.93506	887.9943	9.817798	887.4622	8.623365
	991.2145	16.93769	974.7205	12.80914	986.2928	11.15914	982.5684	10.00405	994.2737	8.883294
Desorption	991.2145	16.93769	974.7205	12.80914	986.2928	11.15914	982.5684	10.00405	994.2737	8.883294
	942.7968	16.80684	946.3881	12.70831	661.6019	10.49443	936.545	9.954897	838.1135	8.803758
	741.1451	16.15967	739.283	12.33208	845.9614	10.8612	859.928	9.798538	747.2639	8.607587
	595.3601	15.68535	534.572	11.54962	564.5005	10.25023	840.5078	9.774439	498.2588	8.07165
	361.9178	15.00073	489.0806	11.35752	598.5524	10.30329	748.062	9.629219	365.7752	7.734469
	295.011	14.61937	361.1197	10.83146	411.0005	9.752666	386.9247	8.656765	300.1985	7.336054
	227.4391	14.2012	299.2675	10.48836	384.2644	9.631785	288.8922	8.199207	264.4174	7.256694
	199.5058	14.02817	258.6977	10.28814	253.3771	9.056412	154.8125	7.072126	197.3775	6.751844
	103.6016	13.15042	199.5058	9.810687	199.5058	8.673324	92.42826	6.678438	179.4204	6.742672
	94.55652	12.98473	94.55652	8.804128	132.5989	8.152355	51.06041	5.893059	116.7701	6.228708
	75.93434	12.65178	68.75147	8.303347	86.57558	7.649366	49.97597	5.789481	94.02442	5.92677
	49.99062	12.21744	49.98147	7.808567	74.95126	7.410664	21.49951	5.268385	56.64708	5.476443
	18.58551	11.25216	28.07278	7.235631	45.94619	6.819636	9.98452	4.815526	49.9247	5.306062
	9.994897	10.76551	19.97964	6.682795	19.98696	6.051057	4.998864	4.345318	18.22782	4.535593
	4.997643	10.12507	11.12351	5.708536	12.99557	5.552403	0.133367	2.580327	9.972923	4.152541
	0.127983	7.366735	9.964988	5.628201	9.995507	5.367493			4.874955	3.615283
			0.115687	5.561354	0.106419	5.284653			0.097562	1.907307

Table S10. Adsorption data for NH_3 in UiO-66-Cu^{I}

Temperature	273K		283K		293K		303K		313K	
Content	Pressure (mbar)	Uptake (mmol/g)	Pressure (mbar)	Uptake (mmol/g)	Pressure (mbar)	Uptake (mmol/g)	Pressure (mbar)	Uptake (mmol/g)	Pressure (mbar)	Uptake (mmol/g)
Adsorption	0.50054	1.303986	0.496976	1.090724	0.50159	2.760388	0.503214	2.285666	0.501493	2.201978
	0.997043	1.718224	0.99664	1.468398	1.000351	2.981177	1.00123	2.553695	0.997739	2.402182
	1.39769	1.950173	1.400022	1.678253	1.40018	3.127827	1.400034	2.645331	1.400131	2.519389
	1.871232	2.145865	1.937154	1.835738	1.895037	3.242598	2.006738	2.776188	1.88344	2.631151
	2.528621	2.279078	2.471854	1.954764	2.548763	3.350252	2.490777	2.873348	2.562192	2.719439
	4.959799	2.688083	4.968345	2.357531	4.995812	3.653099	4.989708	3.144998	4.9775	2.994419
	9.939961	3.178354	9.994287	2.797552	9.977196	4.015043	9.991235	3.509008	9.990013	3.275447
	14.97689	3.552448	14.95553	3.105745	14.95431	4.260971	14.98666	3.764703	14.97384	3.506812
	19.98513	3.872219	19.97414	3.351724	19.95705	4.456861	20.00283	3.929942	19.97475	3.664015
	49.96804	5.242761	49.95461	4.261394	49.93813	5.2181	49.9949	4.585775	49.9479	4.223208
	74.97628	6.222986	74.96957	4.93399	74.96102	5.687066	74.98055	4.951979	74.98422	4.527739
	98.14791	6.852391	98.81299	5.540513	96.81776	6.00973	99.34505	5.283319	98.14791	4.736617
	117.0361	7.329958	121.9577	6.025938	118.4993	6.310622	121.0266	5.489754	118.8984	4.919124
	143.5062	7.935429	145.5015	6.500181	142.043	6.608326	146.5656	5.764409	142.7081	5.096317
	168.2471	8.376575	170.7744	6.910704	165.7198	6.880153	171.7056	5.953558	166.3849	5.273231
	192.323	8.738371	194.7172	7.281187	187.5344	7.113764	195.2493	6.209383	179.1544	5.441982
	243.0018	9.357524	244.332	7.886881	235.819	7.512372	246.3272	6.501386	236.3511	5.720651
	304.588	10.07221	315.2293	8.614859	300.9966	7.994052	316.6925	6.959524	301.1297	6.062601
	371.3619	10.67946	385.3285	9.288923	367.2384	8.430622	389.319	7.296359	394.2406	6.508943
	475.2471	11.15794	480.1686	10.07934	499.5889	8.947686	480.0356	7.692384	495.8645	6.803347

	598.1534	11.47367	580.4623	10.76082	595.8922	9.410358	588.7094	8.094087	600.2817	7.249777
	695.9199	11.79115	673.4402	11.19924	698.8462	9.696459	690.9982	8.491462	693.2595	7.52505
	794.4844	12.05794	774.2661	11.42858	789.9619	9.934404	796.0806	8.729941	795.2825	7.792143
	899.9658	12.33112	875.3578	11.56193	893.98	10.15608	891.8517	9.047915	889.1914	8.019883
	988.155	12.59935	996.269	11.70144	998.5303	10.36898	996.136	9.156348	990.9484	8.214856
Desorption	988.155	12.59935	996.269	11.70144	998.5303	10.36898	996.136	9.156348	990.9484	8.214856
	842.769	12.24884	844.0992	11.5838	844.0992	10.10988	847.8236	8.919154	849.0208	8.014081
	743.0074	12.06842	809.9142	11.47514	594.163	9.647686	753.3826	8.786917	663.3311	7.710523
	591.2366	11.76723	646.7041	11.10836	590.5715	9.589865	615.8445	8.512402	595.4931	7.553676
	529.5174	11.67093	590.7046	10.94373	492.9381	9.397857	597.4883	8.473594	553.1942	7.497223
	400.3593	11.19705	437.0716	10.44542	396.5018	9.043776	447.4468	8.1866	400.3593	7.058849
	246.9923	10.55129	398.4971	10.26885	360.7206	8.973829	380.2739	7.930716	394.6396	7.086999
	230.6314	10.53442	262.4221	9.697526	247.5244	8.41458	289.6903	7.639759	256.9685	6.582795
	157.8719	10.05157	184.076	9.166866	196.7124	8.129366	233.0257	7.295913	243.6669	6.500276
	145.6345	9.909918	141.777	8.755571	146.8316	7.792137	187.1353	7.047763	198.1756	6.273324
	84.58035	9.310926	91.63016	8.210416	84.97939	7.20512	136.1904	6.734856	133.6631	5.916177
	74.93722	9.097601	74.94027	7.887691	74.94943	7.020893	93.75842	6.385766	92.16222	5.537734
	43.38561	8.474217	49.04574	7.293329	45.28697	6.465308	25.00436	4.693985	48.80525	4.676888
	20.00161	7.561412	19.99917	6.487644	19.96987	5.758093	20.00113	4.567824	20.00115	4.247726
	9.991313	6.715131	9.996728	5.742386	9.633547	5.058123	9.995507	4.295913	12.99801	4.051585
	4.996847	6.185465	4.996422	5.159752	4.994591	4.705718	4.995812	3.952604	9.990624	3.904208
	0.154968	5.641685	0.161787	3.314207	0.161506	3.022412	0.136285	2.62018	0.580282	3.110442

Table S11. Adsorption data for NH_3 in UiO-66-defect

Temperature	273K		283K		293K		303K		313K	
Content	Pressure (mbar)	Uptake (mmol/g)	Pressure (mbar)	Uptake (mmol/g)	Pressure (mbar)	Uptake (mmol/g)	Pressure (mbar)	Uptake (mmol/g)	Pressure (mbar)	Uptake (mmol/g)
Adsorption	0.996091	0.899673	1.121184	1.4071	1.108305	1.348594	0.997873	0.882688	0.998434	0.348373
	1.396274	1.022695	1.400046	1.454029	1.401865	1.36531	1.400864	0.945892	1.400168	0.380011
	1.93166	1.15053	1.927388	1.550166	1.891375	1.45098	1.885881	1.022138	1.893206	0.449409
	2.498712	1.252263	2.529841	1.636476	2.539608	1.530957	2.484673	1.094284	2.538387	0.522696
	4.969566	1.523987	4.99215	1.899552	4.970787	1.771063	4.985435	1.318971	4.984215	0.727701
	9.992455	1.832736	9.978416	2.243777	9.959494	2.082653	9.958883	1.617135	9.998559	0.978254
	14.9482	2.104159	14.95431	2.516466	14.99337	2.300885	14.98055	1.813883	14.98788	1.174093
	19.97842	2.312115	19.95888	2.730959	19.95949	2.476312	19.9656	1.966609	19.99734	1.313644
	49.96133	3.416124	49.97842	3.621455	49.94362	3.190157	49.9424	2.565713	49.93569	1.844112
	74.95919	4.231462	74.99703	4.234103	74.92135	3.647041	74.97811	2.921402	74.94698	2.154451
	94.6895	4.936121	97.08381	4.652228	94.95555	3.96616	95.08857	3.185814	95.62063	2.370729
	113.4447	5.452849	117.1692	4.958725	116.105	4.2197	114.1098	3.39032	115.706	2.551058
	142.9742	6.027912	140.9789	5.266985	135.3923	4.452774	134.5942	3.598295	138.9837	2.736817
	171.0405	6.541158	163.5916	5.538345	156.8078	4.673606	154.5465	3.784065	160.9313	2.910642
	184.209	6.891874	182.7458	5.774759	185.9382	4.913626	190.3277	4.006667	182.4798	3.069709
	222.9165	7.542168	225.3108	6.188604	220.6553	5.211022	227.5721	4.254915	226.3749	3.332062
	319.2198	8.961056	300.9966	6.721342	324.5404	5.678899	317.7566	4.803599	305.1201	3.665114
	395.3047	9.799483	395.4377	7.321765	391.9793	6.218788	390.9152	5.168874	398.7631	4.148161
	500.254	10.24458	494.1352	7.717931	496.5296	6.620092	498.9238	5.56513	497.1946	4.536465
	593.6309	10.56792	599.2175	8.055372	593.6309	6.927713	587.9112	5.847299	593.2318	4.843636

	688.471	10.87309	691.5303	8.335521	681.6871	7.171207	688.205	6.092978	693.5256	5.109712
	784.5082	11.17647	787.3016	8.603288	788.8978	7.435099	782.1139	6.324706	784.2422	5.326683
	884.4029	11.4871	889.4575	8.894117	886.6642	7.654057	886.7972	6.541375	898.3696	5.546988
	983.6325	11.78901	986.1598	9.16277	992.5446	7.899888	981.7703	6.749366	984.1646	5.719868
Desorption	983.6325	11.78901	986.1598	9.16277	992.5446	7.899888	981.7703	6.749366	984.1646	5.719868
	849.1538	11.39517	845.6954	8.825487	947.1863	7.826896	848.6217	6.487893	841.0399	5.474888
	749.9242	11.10291	746.8648	8.606083	837.8475	7.636009	587.2461	5.993219	736.2235	5.306677
	586.5811	10.59165	581.3935	8.282034	748.1949	7.446636	464.7388	5.770573	595.2271	5.024015
	495.8645	10.35444	493.0711	7.906957	490.9429	6.823133	395.7037	5.568706	553.5932	4.991966
	304.588	9.397951	448.5109	7.843436	420.3116	6.682451	388.1219	5.449595	393.0434	4.501623
	241.6717	8.800112	314.8303	7.301309	301.7947	6.228981	307.7805	5.253853	350.6115	4.433617
	157.7389	8.11988	201.767	6.668542	208.5508	5.752959	213.0733	4.824969	229.3013	3.955403
	125.1501	7.655478	170.1093	6.416921	192.0569	5.581188	152.1522	4.449666	160.3992	3.585002
	83.2502	7.028558	134.1951	6.089479	127.5444	5.183932	127.6774	4.240513	122.2237	3.341746
	74.9012	6.742919	88.30477	5.592731	82.7181	4.771851	83.51621	3.889682	85.91051	3.048214
	43.53454	5.969076	74.97201	5.319557	74.97262	4.594984	74.97079	3.733382	74.93599	2.891163
	19.99246	5.041827	45.8119	4.725993	45.56225	4.110197	45.38952	3.311662	45.60193	2.49117
	9.60791	4.234445	20.00161	3.995944	19.96071	3.49544	19.9772	2.766563	19.98391	1.987182
	4.996422	3.763741	9.85878	3.337407	9.757455	2.888419	10.15421	2.206758	9.996585	1.584654
	0.172639	2.122122	4.994591	2.937775	4.993981	2.544769	4.995812	1.880834	4.991463	1.121658
			0.173811	1.417481	0.172835	1.143739	0.17408	0.579548	0.121685	0.846554

References:

(1) Ma, Y.; Han, X.; Xu, S.; Wang, Z.; Li, W.; da Silva, I.; Chansai, S.; Lee, D.; Zou, Y.; Nikiel, M.; Manuel, P.; Sheveleva, A. M.; Tuna, F.; McInnes, E. J. L.; Cheng, Y.; Rudić, S.; Ramirez-Cuesta, A. J.; Haigh, S. J.; Hardacre, C.; Schröder, M.; Yang, S. Atomically dispersed copper sites in a metal−organic framework for reduction of nitrogen dioxide. *J. Am. Chem. Soc.* **2021**, 143, 10977−10985.

(2) Abdel-Mageed, A. M.; Rungtaweevoranit, B.; Parlinska-Wojtan, M.; Pei, X.; Yaghi, O. M.; Behm, R. J. Highly active and stable single atom Cu catalysts supported by a metal-organic framework. *J. Am. Chem. Soc.* **2019**, 141, 5201−5210.

(3) Hutter, J.; Iannuzzi, M.; Schiffmann, F.; VandeVondele, J. cp2k: atomistic simulations of condensed matter systems. *Wiley Interdiscip. Rev.: Comput. Mol. Sci.* **2014**, 4, 15–25.

(4) Lippert, G.; Hutter, J.; Parrinello, M. A hybrid Gaussian and plane wave density functional scheme. *Mol. Phys.* **1997**, 92, 477–487.

(5) Vandevondele, J.; Krack, M.; Mohamed, F.; Parrinello, M.; Chassaing, T.; Hutter, J. Quickstep: Fast and accurate density functional calculations using a mixed Gaussian and plane waves approach. *Comput. Phys. Commun.* **2005**, 167, 103–128.

(6) VandeVondele, J.; Hutter, J. Gaussian basis sets for accurate calculations on molecular systems in gas and condensed phases. *J. Chem. Phys.* **2007**, 127, 114105.

(7) Goedecker, S.; Teter, M.; Hutter, J. Separable dual-space Gaussian pseudopotentials. *Phys. Rev. B* **1996**, 54, 1703–1710.

(8) Perdew, J. P.; Burke, K.; Ernzerhof, M. Generalized gradient approximation made simple. *Phys. Rev. Lett.* **1996**, 77, 3865–3868.

(9) Grimme, S.; Antony, J.; Ehrlich, S.; Krieg, H. A consistent and accurate ab initio parametrization of density functional dispersion correction (DFT-D) for the 94 elements H-Pu. *J. Chem. Phys.* **2010**, 132, 154104.

(10) Cheng, Y. Q.; Daemen, L. L.; Kolesnikov, A. I.; Ramirez-Cuesta, A. J. Simulation of inelastic neutron scattering spectra using OCLIMAX. *J. Chem. Theory Comput.* **2019**, 15, 1974−1982.

(11) Höfer, P. et al. Hyperfine sublevel correlation (HYSCORE) spectroscopy: a 2D ESR investigation of the squaric acid radical. *Chem. Phys. Lett.* **1986**, 132, 279–282.

(12) Stoll, S. & Schweiger, A. EasySpin, a comprehensive software package for spectral simulation and analysis in EPR. *J. Magn. Reson.* **2006**, 178, 42–55.

(13) Han, X.; Hong, Y.; Ma, Y.; Lu, W.; Li, J.; Lin, L.; Sheveleva, A. M.; Tuna, F.; McInnes, E. J. L.; Dejoie, C.; Sun, J.; Yang, S.; Schröder, M. Adsorption of nitrogen dioxide in a redox active vanadium metal-organic framework material. *J. Am. Chem. Soc.* **2020**, 142, 15235−15239.

(14) Kim, D. W.; Kang, D. W.; Kang, M.; Lee, J.; Choe, J. H.; Chae, Y. S.; Choi, D. S.; Yun, H.; Hong, C. S. High ammonia uptake of a metal-organic framework adsorbent in a wide pressure range. *Angew. Chem. Int. Ed.* **2020**, 59, 22531–22536.

(15) Rieth, A. J.; Dincă, M. Controlled gas uptake in metal-organic frameworks with record ammonia sorption. *J. Am. Chem. Soc*. **2018**, 140, 3461–3466.

(16) Van Humbeck, J. F.; McDonald, T. M.; Jing, X.; Wiers, B. M.; Zhu, G.; Long, J. R. Ammonia capture in porous organic polymers densely functionalized with Brønsted acid groups. *J. Am. Chem. Soc.* **2014,** 136, 2432–2440.

(17) Glomb, S.; Woschko, D.; Makhloufi, G.; Janiak, C. Metal-organic frameworks with internal urea-functionalized dicarboxylate linkers for SO_2 and NH_3 desorption. *ACS Appl. Mater. Interfaces.* **2017**, 9, 37419–37434.

(18) Chen, Y.; Shan, B.; Yang, C.; Yang, J.; Li, J.; Mu, B. Environmentally friendly synthesis of flexible MOFs $M(NA)_2$ (M= Zn, Co, Cu, Cd) with large and regenerable ammonia capacity. *J. Mater. Chem. A.* **2018**, 6, 9922–9929.
(19) Han, X.; Lu, W.; Chen, Y.; da Silva, I.; Li, J.; Lin, L.; Li, W.; Sheveleva, A. M.; Godfrey, H. G. W.; Lu, Z.; Tuna, F.; McInnes, E. J. L.; Cheng, Y.; Daemen, L. L.; McPherson, L. J. M.; Teat, S. J.; Frogley, M. D.; Rudic, S.; Manuel, P.; Ramirez-Cuesta, A. J.; Yang, S.; Schroder, M. High ammonia adsorption in MFM-300 materials: dynamics and charge transfer in host-guest binding. *J. Am. Chem. Soc.* **2021**, 143, 3153–3161.
(20) Spanopoulos, I.; Xydias, P.; Malliakas, C. D.; Trikalitis, P. N. A straight-forward route for the development of metal-organic frameworks functionalized with aromatic -OH groups: synthesis, characterization, and gas (N_2, Ar, H_2, CO_2, CH_4, NH_3) sorption properties. *Inorg. Chem*. **2013**, 52, 855–862.
(21) Kajiwara, T.; Higuchi, M.; Watanabe, D.; Higashimura, H.; Yamada, T.; Kitagawa, H. A systematic study on the stability of porous coordination polymers against ammonia. *Chem. Eur. J.* **2014**, 20, 15611–15617.
(22) Godfrey, H. G. W.; da Silva, I.; Briggs, L.; Carter, J. H.; Morris, C. G.; Savage, M.; Easun, T. L.; Manuel, P.; Murray. C. A.; Tang, C. C.; Frogley. M. D.; Cinque, G.; Yang, S.; Schröder, M. Ammonia storage by reversible host-guest site exchange in a robust metal-organic framework. *Angew. Chem. Int. Ed.* **2018**, 57, 14778–14781.
(23) Rieth, A. J.; Tulchinsky, Y.; Dincă, M. High and reversible ammonia uptake in mesoporous azolate metal–organic frameworks with open Mn, Co, and Ni sites. *J. Am. Chem. Soc*. **2016**, 138, 9401–9404.
(24) Chen, Z.; Wang, X.; Cao, R.; Idrees, K. B.; Liu, X.; Wasson, M.; Farha, O. K. Water-based synthesis of a stable iron-based metal-organic framework for capturing toxic gases. *ACS Materials Lett.* **2020**, 2, 1129–1134.
(25) Saha, D.; Deng, S.; Ammonia adsorption and its effects on framework stability of MOF-5 and MOF-177. *J. Colloid Interface Sci.* **2010**, 348, 615–620.
(26) Petit, C.; Huang, L.; Jagiello, J.; Kenvin, J.; Gubbins, K. E.; Bandosz, T. J. Toward understanding reactive adsorption of ammonia on Cu-MOF/graphite oxide nanocomposites. *Langmuir.* **2011**, 27, 13043–13051.
(27) Cao, Z.; Landström, K. N.; Akhtar, F. Rapid ammonia carriers for CR systems using MOFs $[M_2(adc)_2(dabco)]$ (M=Co, Ni, Cu, Zn). *Catalysts.* **2020**, 10, 1444–1454.
(28) Moribe, S.; Chen, Z.; Alayoglu, S.; Syed, Z. H.; Islamoglu, T.; Farha, O. K. Ammonia capture within isoreticular metal−organic frameworks with rod secondary building units. *ACS Mater. Lett.* **2019**, 1, 476–480.
(29) Chen, Y.; Zhang, F.; Wang, Y.; Yang, C.; Yang, J.; Li, J. Recyclable ammonia uptake of a MIL series of metal-organic frameworks with high structural stability. *Microporous Mesoporous Mater.* **2018**, 258, 170–177.
(30) Yoskamtorn, T.; Zhao, P.; Wu, X. P.; Purchase, K.; Orlandi, F.; Manuel, P.; Taylor, J.; Li, Y.; Day, S.; Ye, L.; Tang, C. C.; Responses of defect-rich Zr-based metal-organic frameworks toward NH_3 adsorption. *J. Am. Chem. Soc.* **2021**, 143, 3205–3218.
(31) Zhang, Y.; Zhang, X.; Chen, Z.; Otake, K.; Peterson, G. W.; Chen, Y.; Wang, X.; Redfern, L. R.; Goswami, S.; Li, P.; Islamoglu, T.; Wang, B.; Farha, O. K. A Flexible interpenetrated zirconium-based metal-organic framework with high affinity toward ammonia. *ChemSusChem*. **2020**, 13, 1710–1714.
(32) Chen, Y.; Zhang, X.; Ma, K.; Chen, Z.; Wang, X.; Knapp, J.; Alayoglu, S.; Wang, F.; Xia, Q.; Li, Z.; Islamoglu, T.; Farha, O. K. Zirconium-based metal-organic framework with 9-connected nodes for ammonia capture. *ACS Appl. Nano Mater*. **2019**, 2, 6098–6102.
(33) Morris, W.; Doonan, C. J.; Yaghi, O. M. Postsynthetic modification of a metal–organic framework for stabilization of a hemiaminal and ammonia uptake. *Inorg. Chem.* **2011**, 50, 6853–6855.

(34) Liu, J.; Chen, Z.; Wang, R.; Alayoglu, S.; Islamoglu, T.; Lee, S.; Sheridan, T. R.; Chen, H.; Snurr, R. Q.; Farha, O. K.; Hupp, J. T. Zirconium metal-organic frameworks integrating chloride ions for ammonia capture and/or chemical separation. *ACS Appl. Mater. Interfaces.* **2021**, 13, 22485–22494.

(35) Liu, J.; Lu, Z.; Chen, Z.; Rimoldi, M.; Howarth, A. J.; Chen, H.; Alayoglu, S.; Snurr, R. Q.; Farha, O. K.; Hupp, J. T. Ammonia capture within zirconium metal-organic frameworks: reversible and irreversible uptake. *ACS Appl. Mater. Interfaces.* **2021**, 13, 20081–20093.

(36) Sun, L. J.; Fan, Y. L.; Yin, M. J.; Zhang, H. P.; Feng, H.; Guo, L. J.; Luo, F. Thorium metal-organic framework showing proton transformation from $[NH_2(CH_3)_2]^+$ to the carboxyl group to enhance porosity for selective adsorption of D_2 over H_2 and ammonia capture. *Cryst. Growth Des.* **2020**, 20, 3605–3610.

(37) Nguyen, T. N.; Harreschou, I. M.; Lee, J. H.; Stylianou, K. C.; Stephan, D. W. A recyclable metal-organic framework for ammonia vapour adsorption. *Chem. Commun*. **2020**, 56, 9600.

(38) Lyu, P.; Wright, A. M.; López-Olvera, A.; Mileo, P. G.; Zárate, J. A.; Martínez-Ahumada, E.; Martis, V.; Williams, D. R.; Dincă, M.; Ibarra, I. A.; Maurin, G.; Ammonia capture via an unconventional reversible guest-induced metal-linker bond dynamics in a highly stable metal-organic framework. *Chem. Mater.* **2021**. 33, 6186–6192

(39) Marsh, C.; Han, X.; Li, J.; Lu, Z.; Argent, S. P.; Da Silva, I.; Cheng, Y.; Daemen, L. L.; Ramirez-Cuesta, A. J.; Thompson, S. P.; Blake, A. J.; Yang, S.; Schröder, M. Exceptional packing density of ammonia in a dual-functionalized metal–organic framework. *J. Am. Chem. Soc.* **2021**, 143, 6586–6592.

(40) Britt, D.; Tranchemontagne, D.; Yaghi, O. M. Metal-organic frameworks with high capacity and selectivity for harmful gases. *Proc. Natl. Acad. Sci. U.S.A.* **2008**, 105, 11623–11627.
